# Aza-BODIPY-based polymeric nanoparticles for photothermal cancer therapy in a chicken egg tumor model†

**DOI:** 10.1039/d3na00718a

**Published:** 2023-10-27

**Authors:** Kantapat Chansaenpak, Gong Yi Yong, Anawin Prajit, Peraya Hiranmartsuwan, Shaamini Selvapaandian, Bongkot Ouengwanarat, Tunyawat Khrootkaew, Piyanut Pinyou, Chin Siang Kue, Anyanee Kamkaew

**Affiliations:** a National Nanotechnology Center, National Science and Technology Development Agency Thailand Science Park Pathum Thani Thailand 12120 kantapat.cha@nanotec.or.th; b Faculty of Health and Life Sciences, Management and Science University Seksyen 13 Shah Alam Selangor Malaysia 40100 cskue@msu.edu.my; c School of Chemistry, Institute of Science, Suranaree University of Technology Nakhon Ratchasima Thailand 30000 anyanee@sut.ac.th

## Abstract

A new push–pull aza-BODIPY (AZB-CF_3_) derivative comprised of dimethylamino groups and trifluoromethyl moieties was successfully synthesized. This derivative exhibited broad absorption in the near-infrared region in the range from 798 to 832 nm. It also exhibited significant near-infrared (NIR) signals in low-polar solvents with emission peaks around 835–940 nm, while non-fluorescence in high-polar environments due to the twisted intramolecular charge transfer (TICT) phenomenon. The nanoprecipitation of this compound with phospholipid-based polyethylene glycol (DSPE-PEG) yielded AZB-CF_3_@DSPE-PEG nanoparticles (NPs) with a hydrodynamic size of 70 nm. The NPs exhibited good photostability, colloidal stability, biocompatibility, and excellent photothermal (PTT) competence with a conversion efficiency (*η*) of 44.9%. These NPs were evaluated *in vitro* and *in ovo* in a 4T1 breast cancer cell line for NIR light-trigger photothermal therapy. Proven in the chicken egg tumor model, AZB-CF_3_@DSPE-PEG NPs induced severe vascular damage (∼40% vascular destruction), showed great anticancer efficacy (∼75% tumor growth inhibition), and effectively inhibited distant metastasis *via* photothermal treatment. As such, this PTT-based nanocarrier system could be a potential candidate for a clinical cancer therapy approach.

## Abstract 2

Novel aza-BODIPY-based polymeric nanoparticles have been shown in a chicken egg tumor model to be promising photothermal agents for breast cancer treatment.

## Graphical abstract

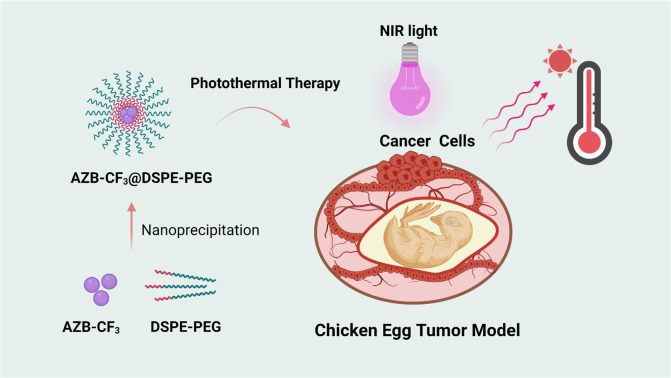

## Introduction

Cancer is still a serious menace to human health among human non-communicable diseases (NCD) despite many methods have been developed to conquer its risk.^1–4^ Due to the fast proliferation of tumor cells, traditional cancer treatments, including radiation therapy, chemotherapy, surgery, cannot cure cancer effectively.^5–7^ Recently, phototherapies, such as photodynamic therapy (PDT) and photothermal therapy (PTT), have received growing attention for tumor ablation owing to their low toxicity, low side effects, and no drug resistance.^8–10^ The PTT technique is a potent and minimally invasive cancer treatment that can induce apoptosis through the elevation of the tissue temperature.^11–13^ The thermal effect is generated artificially when near-infrared (NIR) light is applied in the presence of the PTT agent. The NIR light benefits PTT in terms of high penetration depth and minimal cytotoxicity due to the low photon absorption of endogenous biomolecules in the NIR wavelength.^14–16^ In addition, PTT treatments rely on the administration of tumor-targeting photosensitizers (PS), followed by local irradiation at the tumor location. The formulation of those PS into nanoscale materials can lead to a high accumulation of PS in the tumor sites *via* enhanced permeability and retention (EPR) effects, thus enhancing PTT efficiency.^17–19^

Azadipyrromethene boron difluoride (aza-BODIPY) derivatives are recently developed PS that possess suitable properties for cancer phototherapeutic applications, including near-infrared absorption, high extinction coefficients, and excellent photostability.^20–26^ Most importantly, their absorption range and photothermal conversion efficiency can be nicely tuned through structural modifications of the aza-BODIPY core by varying the electronic-tuning substituents. Recently, our group has successfully introduced the push–pull electronic effect into the aza-BODIPY backbone consisting of electron-donating dimethylamino groups (push moiety), as well as the strong electron-withdrawing cyano group (pull moiety), which can adjust its absorption range to 850–887 nm in various solvents.^27^ Furthermore, adding dimethylamino units to aza-BODIPY has been shown to encourage photothermal conversion as well as significantly decrease fluorescence quantum yield *via* non-radiative relaxation.^27,28^

**Scheme 1:**
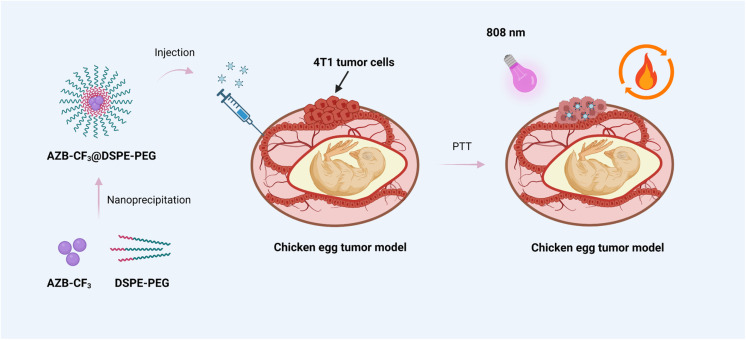
The overall concept of the aza-BODIPY-based polymeric nanoparticles for photothermal cancer therapy in the chicken egg tumor model.

As part of our continued investigation on PTT-based aza-BODIPY, we have synthesized a new push–pull aza-BODIPY analog containing dimethylamino groups and trifluoromethyl groups (AZB-CF_3_). Similar to other aza-BODIPY derivatives in the literature,^29–31^ the hydrophobic AZB-CF_3_ needs to be formulated into nanoparticles to enhance the water suspendability and tumor-targetableity of the compound. In this work, the amphiphilic phospholipid–polymer conjugate, 1,2-distearoyl-*sn-glycero*-3-phosphoethanolamine-poly(ethylene glycol) (DSPE-PEG) was selected as the encapsulating material due to its biocompatibility, biodegradability, and great colloidal stability in biological matrices.^32–34^ After nanoparticle formulation *via* the nanoprecipitation method, the PTT efficiency of the prepared nanoparticles for cancer treatment was then examined in the chicken egg tumor model as demonstrated in Scheme 1. In this model, the tumor cells can be efficiently engrafted on the vascularized chorioallantoic membrane (CAM) due to the natural immunodeficiency of the avian embryo. This *in ovo* model can serve as an alternative for mammalian *in vivo* models to investigate the characteristics of tumor growth, metastasis, angiogenesis, and efficacy of the cancer phototherapies of the nanoparticles.^35–37^

## Experimental section

### Materials and instruments

Boron trifluoride etherate (BF_3_OEt_2_) was purchased from Sigma Aldrich. 1,2-Distearoyl-*sn-glycero*-3-phosphoethanol amine-*N*-[methoxyl-(polyethylene glycol)-2000] (DSPE-PEG_2000_) was purchased from Avanti Polar Lipids, Inc. 4-Dimethylaminoacetophenone, 4-(trifluoromethyl) benzaldehyde, nitromethane (CH_3_NO_2_), triethylamine (Et_3_N), and ammonium acetate (NH_4_OAc) were purchased from Tokyo Chemical Industry (TCI). All the chemicals and solvents were used without further purification. UV-vis absorption spectra were acquired from a Cary Series UV-Vis-NIR spectrophotometer (Agilent Tech, Santa Clara, CA, USA). Emission spectra were obtained using a PTI QuantaMaster 500 Near Infra-Red Photoluminescence System (HORIBA Scientific). The details for UV-Vis-NIR and fluorescence measurements and quantum yield calculations are listed in the SI. Dynamic light scattering (DLS) measurements were performed using the Zetasizer Nano series (Malvern Panalytical). Scanning electron microscopy (SEM) images were obtained using a SU-8030 Field-Emission Scanning Electron Microscope (Hitachi). Transmission electron microscopy (TEM) images were acquired from a JEM- 2100-Plus Transmission Electron Microscope (JEOL). Nuclear magnetic resonance (NMR) spectra were obtained on a Bruker NMR 500 spectrometer operating at 500 and 125 MHz for ^1^H and ^13^C NMR, respectively. Electrospray ionization mass spectra were collected from a Bruker micrOTOF spectrometer.

### Synthetic procedures

#### Synthesis of 1-(4-(dimethylamino)phenyl)-3-(4-(trifluoromethyl) phenyl)prop-2-en-1-one (1)

4-Dimethylaminoacetophenone (3 g, 18.4 mmol), KOH pellets (5.16 g, 92.0 mmol), and 4-(trifluoromethyl) benzaldehyde (2.46 mL g, 18.4 mmol) were dissolved in methanol (100 mL). The reaction mixture was stirred at room temperature for 20 h. The precipitate was filtered, washed with cool MeOH, and dried under reduced pressure and the yellow powder was collected (5.55 g, 94.6%). ^1^H NMR (500 MHz, CDCl_3_) *δ*: 7.93 (d, *J* = 8.8 Hz, 2H), 7.71–7.54 (m, 6H), 6.64 (d, *J* = 9.0 Hz, 2H), 3.01 (s, 6H). ^13^C NMR (125 MHz, CDCl_3_) *δ*: 187.07, 153.5., 140.41, 139.93, 130.86, 128.29, 125.82, 124.59, 110.85, 40.05. HRMS (ESI), *m*/*z* calcd for C_18_H_16_F_3_NNaO ([M + Na]^+^): 342.1076, found: 342.1072.

#### Synthesis of 1-(4-(dimethylamino)phenyl)-4-nitro-3-(4-(trifluoromethyl)phenyl)butan-1-one (2)

A solution of 1 (6.58 g, 20.0 mmol) in methanol (100 mL) was added to CH_3_NO_2_ (16 mL) and Et_3_N (16 mL). Then, the solution was refluxed at 78 °C for 48 h. After cooling to room temperature, the solvent was evaporated under reduced pressure. The reaction was then quenched with water (100 mL) and extracted with dichloromethane (2 × 100 mL). The combined organic layers were washed with deionized water (3 × 100 mL) and dried over anhydrous magnesium sulfate. After organic solvent removal, the brown sticky liquid was obtained (7.46 g, 98.2%) and used without further purification. ^1^H NMR (500 MHz, CDCl_3_) *δ*: 7.69 (d, *J* = 8.9 Hz, 2H), 7.43 (d, *J* = 8.9 Hz, 2H), 7.29 (d, *J* = 7.8 Hz, 2H), 6.49 (d, *J* = 8.9 Hz, 2H), 4.73 (dd, *J* = 12.8, 5.8 Hz, 1H), 4.56 (dd, *J* = 12.7, 9.0 Hz, 1H), 4.16 (dt, *J* = 13.3, 6.8 Hz, 1H), 3.02 (m, 2H), 2.88 (s, 6H). ^13^C NMR (125 MHz, CDCl_3_) *δ*: 194.01, 153.80, 144.05, 130.28, 129.87, 129.61, 128.10, 125.85, 125.82, 125.16, 124.08, 110.72, 79.23, 40.39, 39.80, 39.41. HRMS (ESI), *m*/*z* calcd for C_19_H_19_F_3_N_2_NaO_3_ ([M + Na]^+^): 403.1240, found: 403.1238.

#### Synthesis of 4-(2-((5-(4-(dimethylamino)phenyl)-3-(4-(trifluoromethyl)phenyl)-1*H*-pyrrol-2-yl)imino)-3-(4-(trifluoromethyl)phenyl)-2*H*-pyrrol-5-yl)-*N*,*N*-dimethylaniline (AZB-CF_3_)

NH_4_OAc (38.97 g, 0.5 mol) was added to the solution of 2 (8.46 g, 22 mmol) in butanol (100 mL). The reaction mixture was then refluxed at 90 °C for 15 h. After cooling to room temperature, the solution was kept at 4 °C overnight to precipitate a dark green powder. After filtration, the obtained solid was washed with cold water (2 × 50 mL) and cold ethanol (2 × 50 mL) and dried under reduced pressure (3.45 g, 56.7%). The resulting dark green solid (135.1 mg, 0.20 mmol) was then added to a solution mixture of dry toluene (4 mL) and triethylamine (3.65 mL, 26 mmol) under a nitrogen atmosphere. After being stirred for 30 min, boron trifluoride etherate (BF_3_–OEt_2_) (5.46 mL, 44 mmol) was added to the mixture. The solution was further stirred at room temperature for 1 h. The reaction was then quenched with water (30 mL) and extracted with dichloromethane (2 × 50 mL). The combined organic layers were washed with deionized water (2 × 50 mL) and dried over anhydrous sodium sulfate. After organic solvent removal, the residue was purified by flash column chromatography on silica gel eluting with hexane/ethyl acetate (3 : 1–1 : 1) to give the AZB-CF_3_ as a dark purple powder (40.6 mg, 28.3%). ^1^H NMR (500 MHz, DMSO-d_6_) *δ*: 8.43 (d, *J* = 8.1 Hz, 4H), 8.30 (d, *J* = 9.2 Hz, 4H), 7.99 (d, *J* = 8.3 Hz, 4H), 7.85 (s, 2H), 6.97 (d, *J* = 9.2 Hz, 4H), 3.20 (s, 12H). ^13^C NMR (125 MHz, DMSO-d_6_) *δ*: 154.61, 152.09, 144.26, 136.88, 136.28, 132.48, 131.90, 130.43, 129.08, 128.75, 128.60, 125.41, 120.14, 117.44, 116.90, 112.07, 111.90, 111.74, 29.28, 28.97. HRMS (ESI), *m*/*z* calcd for C_38_H_31_BF_3_N_4_O_2_ ([M + H]^+^): 719.2429, found: 719.2444.

### Electrochemical characterization

A three-electrode setup with a PalmSens 4 potentiostat/galvanostat (PalmSens, Houten, the Netherlands) and PSTrace 5.9 software was used to investigate the electrochemical behavior of AZB-CF_3_. The three-electrode setup included a Pt-disk working electrode (3 mm diameter), an Ag/Ag^+^ non-aqueous reference electrode (0.01 M AgNO_3_), and a Pt plate counter electrode. Under an argon-saturated atmosphere, the measurements were carried out by dissolving the compound in dichloromethane with 0.1 M tetrabutylammonium hexafluorophosphate (TBAPF_6_) as a supporting electrolyte.

### Theoretical calculations

The ground state and the first singlet excited state geometries were optimized using the density functional theory (DFT) and the time-dependent (TD) methods. Both methods were applied with the B3LYP hybrid functional and 6-311G basis set (abbreviated B3LYP/6-311G). All the calculations were performed using the TURBOMOLE software package.

### Preparation of AZB-CF_3_@DSPE-PEG NPs

The AZB-CF_3_@DSPE-PEG NPs were prepared by the nanoprecipitation method adapted from the literature.^38^ A THF solution (2 mL) containing phospholipid-poly(ethylene glycol) hybrid polymer, DSPE-PEG_2000_, (1.5 mg), and three different amounts of AZB-CF_3_ (0.6, 0.8, and 1.0 mg) was added to de-ionized (DI) water (20 mL) under continuous stirring. The solution mixture was sonicated for 5 min using a sonicator (CREST) before being uninterruptedly stirred overnight to remove the organic solvent (THF). The NP suspension was filtered through a 0.22 μm filter resulting in a deep purple AZB-CF_3_@DSPE-PEG suspension. The resulting NP aqueous solutions were freeze-dried and stored at 4 °C for further use. The detailed protocols for the determination of encapsulation efficiency (%EE), dye loading percentage, and colloidal stability of the NPs are displayed in the (ESI†).

### Bioapplications of AZB-CF_3_@DSPE-PEG NPs

#### Cell line and cell culture conditions

Murine mammary carcinoma cells (4T1) were maintained in RPMI 1640 complete media with 10% FBS at 37 °C in a humidified atmosphere with 5% CO_2_.

#### Phototoxicity

Approximately 7 × 10^3^ cells per well of 4T1 were seeded on 96-well plates and incubated in complete media for 24 h. All cells were treated with various concentrations of AZB-CF_3_@DSPE-PEG or AZB-CF_3_ (0, 5, 10, and 20 μM) for 6 h before irradiation with an 808 nm laser for 5 min (0.7 W cm^−2^), then culturing was continued in the dark for another 24 h. The cells were then treated with MTT solution (0.5 mg mL^−1^) for 3 hours after being rinsed three times with PBS. The resulting formazan product was then dissolved in DMSO to determine the number of active cells by recording the UV-vis absorbance at 560 nm. The % viability was used to determine relative cell vitality, which was calculated using the formula 100 × (*A*_control_ − *A*_sample_); where *A* = absorbance at 560 nm.

#### Live/dead and apoptosis detection by flow cytometry

Approximately 1 × 10^6^ cells per well of 4T1 were seeded in a 6-well plate for 24 h. After that, the cells were treated with 20 μM of AZB-CF_3_@DSPE-PEG for 6 h. The cells were then washed twice with 0.01 M of PBS buffer (pH 7.4) and irradiated with an 808 nm laser for 5 min. The cells were trypsinized and washed three times with cold 0.01 M PBS buffer (pH 7.4) by centrifugation at 4000 rpm at 4 °C for 3 min and resuspended in 1× Annexin binding buffer (Thermo Fisher Scientific). The cells were incubated with Annexin V fluorescein conjugate (FITC annexin V, Thermo Fisher Scientific) at room temperature for 15 min before adding propidium iodide (PI, Thermo Fisher Scientific) on ice. For LIVE/DEAD staining, the cells were resuspended with PBS containing Calcein AM and PI. Then, 1 × 10^4^ events were analyzed by flow cytometry using an Attune NxT Flow Cytometer (Thermo Fisher Scientific).

#### Chick embryo chorioallantoic membrane model

Fertilized Lohmann brown chicken eggs were purchased from Hong Hing Sdn Bhd, Selangor, Malaysia. The eggs were disinfected with 70% ethanol and incubated following the procedure as established.^39^ On egg developmental day (EDD)-10, the viability of the embryo and macroscopic inspection of the vasculature of the chorioallantoic membrane (CAM) were conducted and the eggs were randomly selected for the study. The *in ovo* study was conducted, not exceeding EDD-17 of incubation to avoid ethical restrictions.

#### Acute toxicity of NPs on chick embryos (*in ovo* toxicity)

AZB-CF_3_@DSPE-PEG NPs and DSPE-PEG were dissolved in normal saline while AZB-CF_3_ was dissolved in a cocktail of 50% cremophor EL and 50% ethanol. All dissolved compounds were further diluted with normal saline to the desired dose at a final volume of 20 μL for administration. The *in ovo* acute toxicity of the NPs was determined on EDD-10. AZB-CF_3_@DSPE-PEG NPs, AZB-CF_3_, and DSPE-PEG at three selected doses of 100, 500 and 1000 μg mL^−1^ were administered intravenously into the chick embryo using a microliter capillary syringe with a 33-gauge needle (*n* = 5 per dose at 20 μL per embryo). The mortality rate was monitored at 24 h post-administration.

#### *In ovo* anti-angiogenesis

On EDD-10, the nontoxic dose of AZB-CF_3_@DSPE-PEG NPs at 20 μg per embryo (1000 μg mL^−1^), with an equivalent dose of DSPE-PEG (12 μg per embryo; 600 μg mL^−1^) and AZB-CF_3_ (8 μg per embryo; 400 μg mL^−1^), was intravenously administered to the chick embryos at 20 μL per embryo (*n* = 5 per group). A disinfected O-ring was placed on the CAM for spot identification post-administration. At 2 min post-administration, photoirradiation was done with an 808 nm laser at 1.0 W cm^−2^ at the targeted blood vessel for 60 s. The vasculature of CAMs was photographed under a stereomicroscope at 10 min post-PTT. Quantification of the blood vessels was done pre- and 10 min post-PTT to determine the percentage of vasculature destruction using Image-J.

#### Grafting of 4T1 tumor cells in CAM and *in ovo* PTT

Murine 4T1 mammary carcinoma cells suspension (2.5 × 10^5^ cells per embryo) in culture media were prepared with growth factor reduced Matrigel (8.9 mg mL^−1^) in a ratio of 1 : 1. The cell mixture at the volume of 25 μL per embryo was loaded on the targeted CAM (EDD-10) enriched with the blood vessels. The eggs were sealed with parafilm and returned to the incubator post-tumor implantation. On EDD-14, the tumor mass grown on CAMs was monitored and randomly selected for the antitumor study. AZB-CF_3_@DSPE-PEG NPs, DSPE-PEG, and AZB-CF_3_ (equivalent dose as described above) were intravenously administered into the chick embryos at 20 μL per embryo (*n* = 5 per group). At 2 min post-administration, tumor tissue was irradiated with an 808 nm laser at 1.0 W cm^−2^ for 60 s. The real-time temperature at the irradiated tumor site was monitored throughout the 60 s of photo-irradiation by using the IR Thermal Imager. The tumor volume was measured using calipers at 24, 48, and 72 h post-PTT. The tumor volume (mm^3^) was calculated according to the equation of volume [(tumor width)^2^ × tumor length/2]. The chick embryos were euthanised under hypothermic conditions (4 °C) for histopathological analysis.

#### Histopathology analysis

Histopathological tissues were prepared following the procedure as established.^40^ On EDD-17, the tumor, liver, and lung tissues were harvested and fixed in 10% neutral buffered formalin, followed by dehydration in ascending concentrations of ethanol (70%, 90%, and 100%). The dehydrated tissues were cleared in xylene and embedded in paraffin. The tissue blocks were cut into 5 μm thick sections and stained with hematoxylin and eosin.

#### Statistical analysis

All data were compared and analyzed using the one-way ANOVA (IBM SPSS version 26). LD_50_ was determined using the SPSS probit analysis. *p* < 0.05 is considered statistically significant.

## Results and discussion

### Synthesis and photophysical properties of AZB-CF_3_

**Scheme 2:**
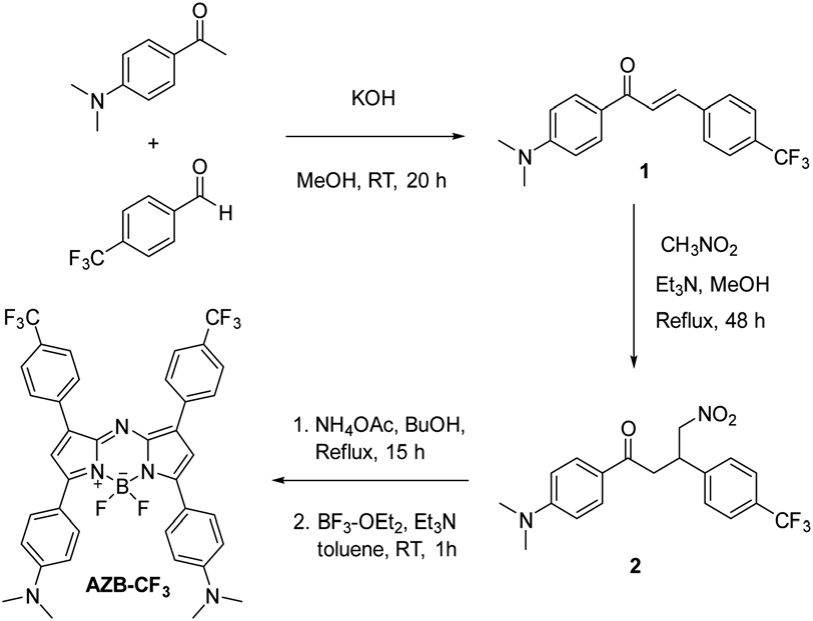
The synthesis of AZB-CF_3_.

AZB-CF_3_ was synthesized *via* the conventional synthetic methods for aza-BODIPY as demonstrated in Scheme 2.^41–43^ In the procedure, the chalcone derivative 1 was simply prepared from an aldol condensation reaction between 4-(trifluoromethyl)benzaldehyde and 4-dimethylaminoacetophenone. The resulting chalcone was then converted to the γ-nitro-substituted ketone product (2) through the Michael addition of nitromethane. Next, compound 2 was reacted with an excess amount of ammonium acetate followed by BF_2_-complexation, yielding AZB-CF_3_ as the final product. The structures of AZB-CF_3_ and its intermediates were confirmed by ^1^H and ^13^C NMR spectroscopic techniques as displayed in the NMR spectra (Fig. S1–S6) in the ESI.† The detection of the molecular ions by positive-mode electrospray mass spectrometry at *m*/*z* = 342.1074, 403.1238, and 719.2444 additionally confirmed the identities of 1, 2, and AZB-CF_3_, respectively (Fig. S7–S9†).

**Fig. 1:**
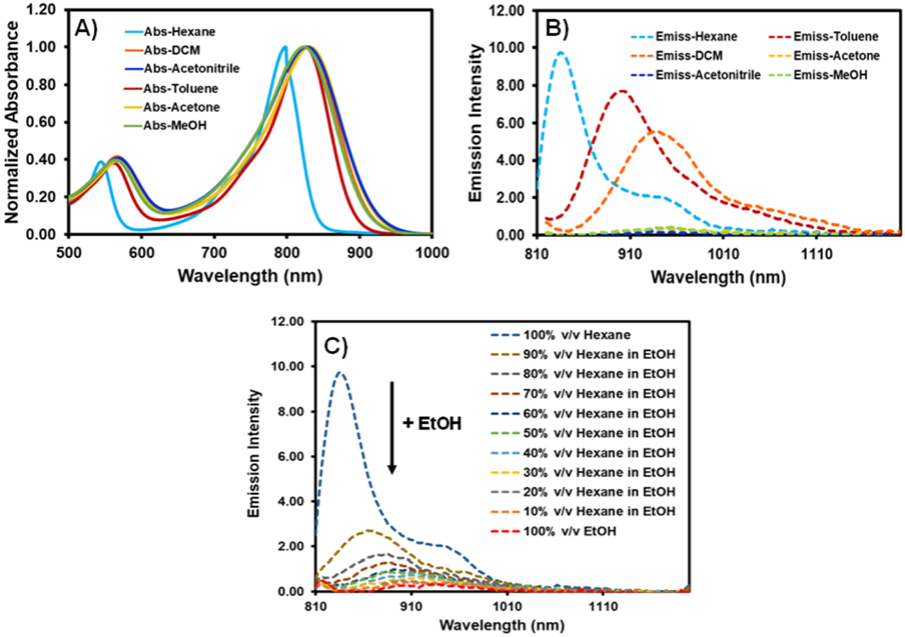
Absorption (A) and emission (B) spectra of AZB-CF_3_ in various solvents and (C) emission spectra of AZB-CF_3_ in hexane–EtOH mixtures (excitation wavelength = 810 nm).

As indicated in Table 1 and Fig. 1A and B, the photophysical characteristics of AZB-CF_3_ were examined using a Vis-NIR and fluorescence spectrophotometer in different solvents. The near-infrared region of AZB-CF_3_ showed wide absorption with a peak at a wavelength between 798 and 832 nm. Interestingly, the dye showed strong fluorescence signals in low-polar solvents with emission maxima at 835, 901, and 937 nm in hexane, toluene, and dichloromethane (DCM), respectively. In contrast to its cyano-analogue,^27^AZB-CF_3_ showed higher fluorescence quantum yields (*Φ*_f_) in low-polar solvents (hexane and toluene) and variable Stokes shifts ranging from 37 to 107 nm. AZB-CF_3_ exhibited weak fluorescence signals in polar solvents, including, acetone, acetonitrile, and methanol, indicating a twisted intramolecular charge transfer (TICT) phenomenon upon excitation.^44,45^ As the concentration of EtOH was increased in the hexane-EtOH systems, AZB-CF_3_ likewise displayed a red shift in emission maxima as well as decreased fluorescence, confirming the molecule's solvatochromic nature as a result of the TICT effect (Fig. 1C).

**Table 1:** Photophysical properties of AZB-CF_3_ (4 uM)

Solvent^a^	*λ*_max_ (nm)	*ε* (M^−1^ cm^−1^)	*λ*_emiss_ b (nm)	Δ*λ* (nm)	*Φ*_f_ c
Hexane	798	7.4 × 10^4^	835	37	0.13 (±0.02)
Toluene	826	8.3 × 10^4^	901	75	0.14 (±0.02)
DCM	830	8.4 × 10^4^	937	107	0.04 (±0.01)
Acetone	832	6.8 × 10^4^	NF^d^		
Acetonitrile	828	6.3 × 10^4^	NF		
MeOH	824	6.3 × 10^4^	NF		

a DCM = dichloromethane, MeOH = methanol.

b The solutions were excited at 810 nm.

c Relative indocyanine green (ICG) in DMSO (*Φ*_f_ = 0.13).

d NF = no fluorescence.

### Electrochemical properties and computational studies of AZB-CF_3_

The electronic properties of AZB-CF_3_ were studied by the cyclic voltammetry (CV) technique. As depicted in Fig. S15,† the cyclic voltammogram of AZB-CF_3_ exhibits two distinct reversible reduction waves for the cathodic scan (−0.65 and −1.20 V) attributed to the one-electron transfer process from the formation of stable radical anions and di-anions.^27^ For the anodic scan, two oxidation peaks were observed. The first oxidation wave at 0.28 V is associated with a one-electron oxidation process, yielding the cation radical. The second oxidation wave coud be attributed to the unstable oxidized form from the first oxidation, which undergoes one-electron oxidation.^46^

Next, the electrochemical bandgap was determined by calculating the first oxidation and reduction potentials obtained from the CV. These potentials provide the energy levels for the highest occupied molecular orbital (HOMO) and lowest unoccupied molecular orbital (LUMO) with reference to the energy levels of the Fc/Fc^+^ redox couple (−4.8 eV relative to the vacuum level). The HOMO and LUMO levels of AZB-CF_3_ can be calculated using the half-wave potential (*E*_1/2_) of Fc/Fc^+^ (0.26 V), as presented in Table 2. The small energy gap of 0.93 eV between the LUMO and HOMO obtained from CV is consistent with the absorption and emission spectra of AZB-CF_3_, which are aligned in a NIR region.

**Table 2:** Electrochemical redox data for reduction potentials, oxidation potentials, and the corresponding LUMO and HOMO levels, and Δ*E*_g_ of AZB-CF_3_^a^

*E*_red2_ (V)	*E*_red1_ (V)	*E*_ox1_ (V)	*E*_ox2_ (V)	LUMO (eV)	HOMO (eV)	Δ*E*_g_ (eV)
−1.20	−0.65	0.28	0.47	−3.89	−4.82	0.93

a *E*_LUMO_ = −4.8 + [*E*_1/2_(Fc) − *E*_red_] eV, *E*_HOMO_ = −4.8 + [*E*_1/2_(Fc) − *E*_ox_] eV, Δ*E*_g_ = *E*_LUMO_ − *E*_HOMO_.

**Fig. 2:**
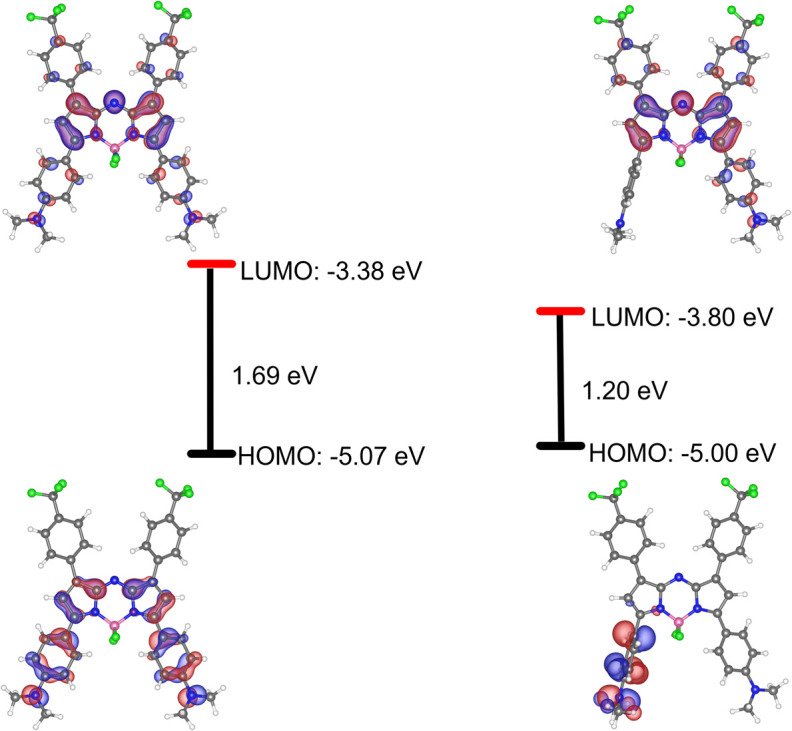
Optimized structures and frontier molecular orbitals (HOMO and LUMO plotted at an electron density value of 0.035) of AZB-CF_3_ in ground states (left) and excited states (right) and their corresponding energy gaps (Δ*E* in eV) computed at the B3LYP/6-311G level of theory.

The electronic phenomenon of this aza-BODIPY derivative was further investigated by density functional theory (DFT) and time-dependent (TD) DFT calculations using the B3LYP/6-311G level of theory. The optimized structures, as well as frontier molecular orbitals (HOMO/LUMO) of AZB-CF_3_ in both the ground state and excited state, are demonstrated in Fig. 2. In the excited state, AZB-CF_3_ demonstrated obvious electron cloud migration from the dimethylaminophenyl moiety in the HOMO to the aza-BODIPY backbone in the LUMO, while their planes were arranged perpendicular to each other, indicating the TICT state.^47^ The low energy gap of 1.20 eV obtained from this calculated method agrees well with that acquired from the experimental method (0.93 eV).

### Preparation and characterizations of AZB-CF_3_ nanoparticles

**Fig. 3:**
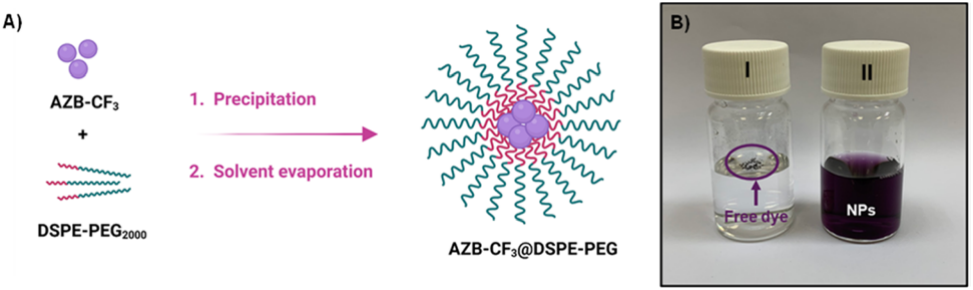
(A) Scheme showing the preparation of AZB-CF_3_@DSPE-PEG NPs, (B) vials containing free dye AZB-CF_3_ (I) and AZB-CF_3_@DSPE-PEG NPs (II) suspended in PBS.

Due to the undesirable hydrophobicity of the aza-BODIPY derivative, AZB-CF_3_ was formulated into nanoparticles (NPs) using 1,2-distearoyl-*sn-glycero*-3-phosphoethanolamine-*N*-[methoxyl-(polyethylene glycol)-2000] (DSPE-PEG), the phospholipid-based PEG polymer, by the nanoprecipitation method (Fig. 3A). To prepare the water-suspendable AZB-CF_3_ NPs (AZB-CF_3_@DSPE-PEG), a THF solution of AZB-CF_3_ (0.6, 0.8, or 1.0 mg) and DSPE-PEG (1.5 mg) was slowly added to 20 mL water under continuous stirring. The mixture was sonicated for 5 min before being stirred overnight to remove THF. The resulting purple suspension was then passed through a 0.22 μm filter yielding AZB-CF_3_@DSPE-PEG NPs. The dried NPs can be thoroughly suspended in a phosphate buffer solution (PBS, pH 7) as shown in Fig. 3B (vial ii), which is different from the free dye (vial i), suggesting the great water-suspendability of the AZB-CF_3_-encapsulated nanomaterials.

**Fig. 4:**
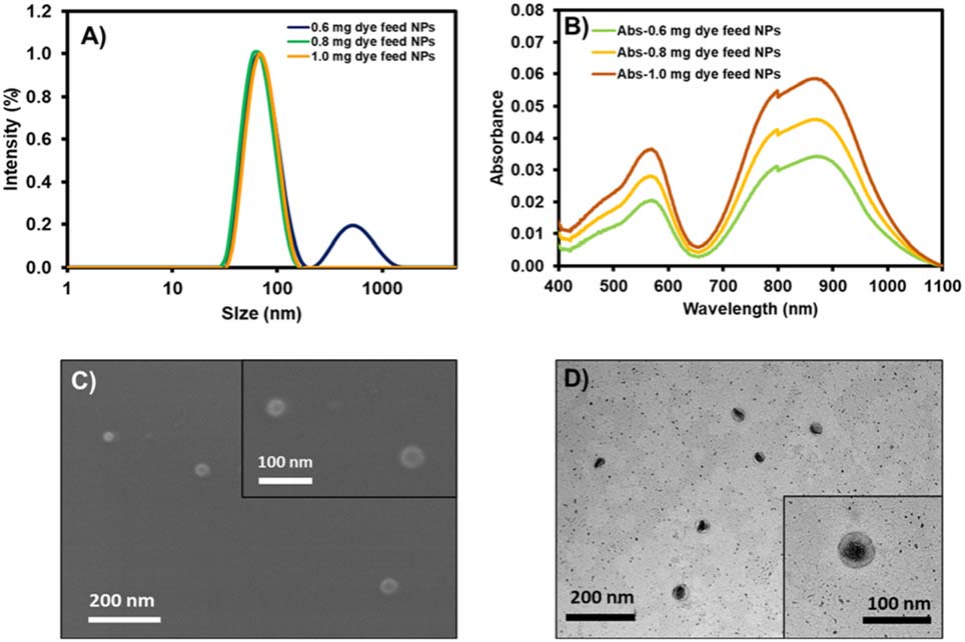
(A) Dynamic light scattering (DLS): the intensity-based size distribution of AZB-CF_3_@DSPE-PEG NPs with 0.6 mg, 0.8 mg, and 1.0 mg of dye feed. (B) Absorption spectra of AZB-CF_3_@DSPE-PEG NPs with different amounts of dye feed. (C) Scanning electron microscope (SEM) images and (D) transmission electron microscope (TEM) images of AZB-CF_3_@DSPE-PEG NPs with 1.0 mg dye feed.

The physical characteristics of NPs were investigated by dynamic light scattering (DLS), scanning electron microscopy (SEM), and transmission electron microscopy (TEM). In the DLS size distributions, the NPs with 0.6 mg of dye feed displayed two groups of size distributions yielding an average size of 87.7 ± 3.1 nm (Fig. 4A and Table 3). The larger size distribution could be assigned to the excess of DSPE-PEG residue, which did not involve dye encapsulation. When the amounts of dye feed were increased to 0.8 and 1.0 mg, only a size distribution peak was observed at 70.0 ± 0.7 nm and 71.7 ± 2.4 nm for the NPs with 0.8 and 1.0 mg of dye feed, respectively. SEM demonstrated the spherical-shaped morphology of the prepared nanoparticles, while TEM revealed the core–shell architecture of the NPs consisting of a dense lyophobic aza-BODIPY core and a soft lyophilic PEG shell (Fig. 4C and D). The absorption spectra of AZB-CF_3_@DSPE-PEG NPs showed a broad absorption band peaking at 870 nm, which agrees well with the absorption spectra of the free AZB-CF_3_, while the intensities of the absorption peak are directly proportional to the amounts of dye feed (Fig. 4B). The AZB-CF_3_@DSPE-PEG NPs, on the other hand, showed no fluorescence in PBS, demonstrating non-radiative relaxation *via* the photothermal effect. This PTT event could be caused by intramolecular charge transfer (ICT) from an electron-donating group, NMe_2_, to the aza-BODIPY center, which serves as the electron-accepting component.^40,48–50^

**Table 3:** The characteristics of AZB-CF_3_-based nanoparticles using different amounts of dye feed^a^

Amount of dye feed (mg)	% Dye loading^b^ (*n* = 3)	% EE^c^ (*n* = 3)	DLS size (nm) (*n* = 3)	PDI (*n* = 3)	Zeta (*ζ*) potential (mV) (*n* = 3)
0.6	27.4 (±0.4)	94.1 (±1.8)	87.7 (±3.1)	0.351 (±0.028)	−5.81 (±0.86)
0.8	33.8 (±0.7)	95.8 (±2.9)	70.0 (±0.7)	0.250 (±0.029)	−5.88 (±1.27)
1.0	39.3 (±0.5)	97.1 (±2.3)	71.7 (±2.4)	0.214 (±0.030)	−5.85 (±0.83)

a Characteristics in each entry were derived from three different batches of nanoparticle preparation (*n* = 3).

b Dye-loading percentages were obtained from the (mass of AZB-CF_3_ found in dried nanoparticles/total mass of dried nanoparticles) × 100.

c Encapsulation Efficiencies (EE) were calculated from the (amount of dye encapsulated in nanoparticle/amount of dye feed) × 100.

The additional characteristics of the AZB-CF_3_-based nanoparticles are demonstrated in Table 3. The lower polydispersity indices (PDIs) of 0.250 and 0.214 were observed in the NPs with 0.8 and 1.0 mg dye feed as only one size distribution curve was detected. The low negative values of zeta-potentials for all conditions (from −5.81 to −5.88 mV) suggested the non-ionic nature of the lipid-based PEG polymeric materials. The great encapsulation efficacies (%EE) of 94.1–97.1% indicated the good efficiency of this nanoprecipitation method, which could hold up to 39.3% of the AZB-CF_3_ dye within the NPs. Next, the AZB-CF_3_@DSPE-PEG NPs with 1.0 mg of dye feed were selected for further studies on photothermal efficacy, colloidal stability, and cancer treatment applications due to their highest photosensitizer content.

**Fig. 5:**
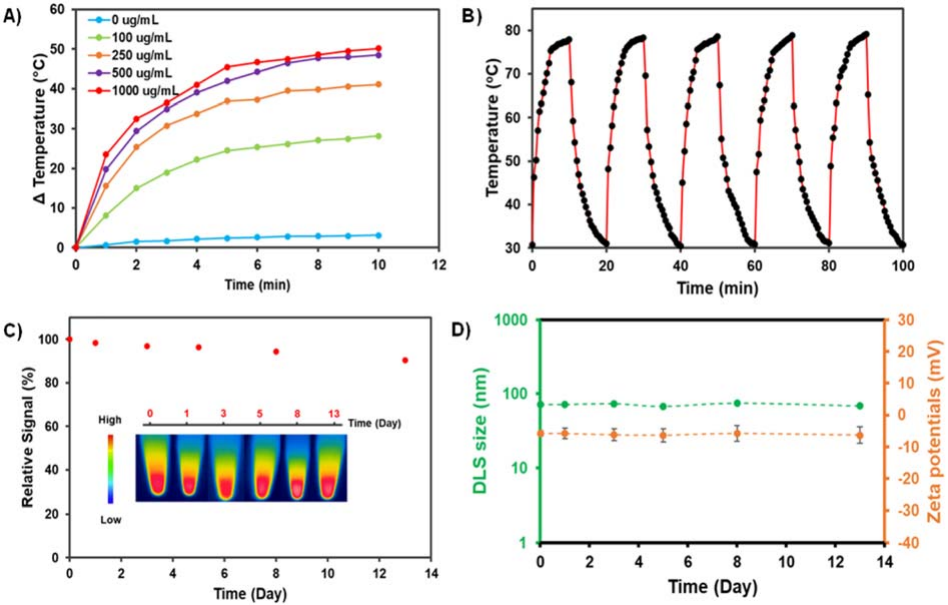
(A) Temperature increase curves of AZB-CF_3_@DSPE-PEG solutions at different NP contents (1,000, 500, 250, and 100 μg mL^−1^) in DI-water after being irradiated by an 808 nm laser with a power density of 1.0 W cm^−2^. (B) Photothermal stability testing with alternate heating and cooling cycles of AZB-CF_3_@DSPE-PEG solution with 1000 μg mL^−1^ of NP content. (C) Absorbance stability of AZB-CF_3_@DSPE-PEG (1.0 mg dye feed) over time. Inset: photograph of heat stability of the AZB-CF_3_@DSPE-PEG. (D) Size (green line) and zeta potential (orange line) of AZB-CF_3_@DSPE-PEG NPs (1.0 mg dye feed) after incubation in 0.1 M phosphate buffer solution (PBS), pH 7.4, for 1, 3, 5, 8, and 13 days (*n* = 5).

To investigate the photothermal effect of the AZB-CF_3_@DSPE-PEG NPs, 1.0 mL of NP suspension in DI water was exposed to 808 nm laser radiation (1.0 W cm^−2^) by varying the irradiation periods (1 to 10 min) and nanoparticle contents (100–1000 μg mL^−1^). As shown in Fig. 5A, the temperature of the AZB-CF_3_@DSPE-PEG in aqueous solutions increased with extended irradiation times in a dose-dependent manner. The NP solution at 1000 μg mL^−1^ exhibited the temperature alteration (Δ*T*) of 50 °C within 10 min of irradiation, indicating excellent photothermal properties. Next, we examined the photostability of the AZB-CF_3_@DSPE-PEG NPs by continuing ON/OFF cycles of irradiation. As displayed in Fig. 5B, the AZB-CF_3_ NPs still sustained a high photothermal effect after five ON/OFF cycles. There were no significant changes in absorbance intensity or the heat signal of the NPs over 13 days (Fig. 5C). After laser irradiation, the size and zeta potential of AZB-CF_3_@DSPE-PEG were assessed to investigate the thermal stability of the particles in contrast to the NPs before irradiation. The results indicated that the structural integrity of the NPs was preserved during photothermal activity based on their size and zeta potential in cell culture media and serum (Fig. S12†). Furthermore, the photothermal conversion efficiency of the nanoparticles was calculated as 44.9% (Fig. S11†), which is comparable to those of other reported aza-BODIPY-based photothermal agents (Table S1†).^22,23,40,51–53^ The generation of singlet oxygen by AZB-CF_3_@DSPE-PEG NPs in an aqueous solution was also investigated. Using ICG as a standard, the results revealed that AZB-CF_3_@DSPE-PEG has a singlet oxygen quantum yield of 0.17 (Fig. S10†). This implies that only photothermal conversion is an important part of phototherapy in this work.

Understanding the colloidal stabilities of the NPs in different biological environments is critical for the further clinical translation of AZB-CF_3_@DSPE-PEG nanomaterials. To demonstrate the colloidal and chemical stabilities of our NPs, we used four different aqueous solutions, including DI water, phosphate-buffered saline (PBS) at pH = 5 and pH = 7, Roswell Park Memorial Institute (RPMI) 1640 culture medium, and fetal bovine serum (FBS). Water and PBS solutions are the most commonly used aqueous solutions in biological research because PBS has the same osmolarity and ion concentrations as the human body. Furthermore, the cell culture medium is dependent on cell type; RPMI-1640 is one of the most common cell culture media that we used to grow 4T1 cells. FBS is also the most widely used growth supplement for cell culture media due to its high content of embryonic growth factors. As shown in Fig. S13,† when dispersed in DI, RPMI, and FBS, AZB-CF_3_@DSPE-PEG demonstrated comparable stability behaviors for up to 24 h to those in PBS at pH = 5 and pH = 7, where the size was slightly larger. This could be due to the competitive binding of DSPE-PEG and phosphate saline. In polymer-stabilized nanoparticle systems, such competitive association and dissociation are common.^54^ When the NPs were dispersed in different solutions for up to 24 h, the Vis-NIR absorbance did not vary considerably, indicating that the dyes inside the particles were stable. The colloidal stability of the NPs was evaluated in 0.1 M PBS at pH = 7.4, for a longer incubation time. As seen in Fig. 5D, the DLS sizes and zeta-potentials of the NPs were negligibly changed in the medium up to day 13 of incubation, suggesting high colloidal stability.

### Photothermal efficiency of AZB-CF_3_ nanoparticles *in vitro*

**Fig. 6:**
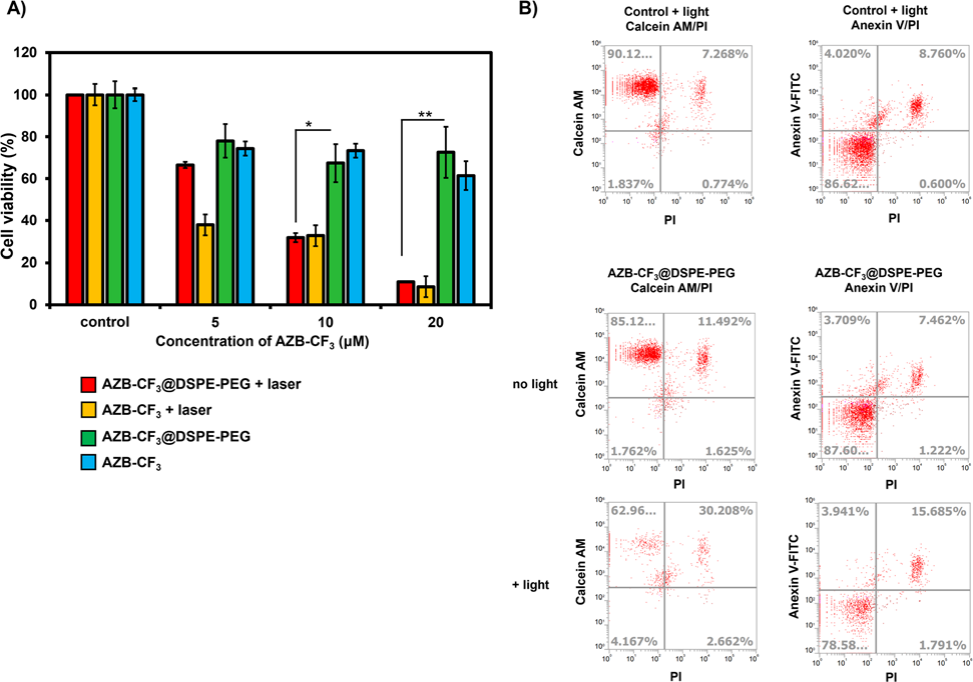
(A) The relative cell viability of 4T1 with and without 808 nm laser irradiation for 5 min (0.7 W cm^−2^) after incubation with AZB-CF_3_@DSPE-PEG or AZB-CF_3_ (0–20 μM) for 6 h. (B) Flow cytometry Annexin V fluorescein isothiocyanate (FITC)/propidium iodide (PI) apoptosis and Calcein AM/PI LIVE/DEAD analyses. Statistical analysis is based on student's *t*-test (^*^P < 0.05, ^**^P < 0.01) as compared to the non-irradiated group.

To demonstrate the efficacy of AZB-CF_3_@DSPE-PEG in cancer cell treatment *via* PTT, the murine mammary carcinoma cells (4T1) were treated with the NPs under light irradiation in comparison to the free dye (AZB-CF_3_) treatment, and their viability was assessed using the MTT assay.^55^ As shown in Fig. 6A, cell viability decreased as the concentration of AZB-CF_3_@DSPE-PEG or AZB-CF_3_ increased when exposed to 808 nm laser irradiation for 5 min (1.0 W cm^−2^). At 20 μM, light exposure reduced cell viability to approximately 10 percent, which is significantly lower than without laser illumination. However, at high doses, NPs and free dye exhibited some dark toxicity.

Calcein-AM and propidium iodide (PI) staining were used to identify viable and dead cells. When calcein-AM enters live cells, it emits green fluorescence after being cleaved by intracellular esterase, whereas PI only interacts with dead cell nuclei and emits red fluorescence. As shown in Fig. 6B, after incubating cells with AZB-CF_3_@DSPE-PEG followed by light irradiation, many dead cells were observed from 4T1, while there were fewer live cells, with only 63% of live cells existing after light irradiation, where the percentage of dead cells was up to 30%. When compared to cells treated with NPs without light irradiation, viable and dead cells were 85 and 11%, respectively.

We used flow cytometry to investigate the photothermal-assisted apoptosis of cancer cells by our NPs. To identify apoptotic cells, an apoptosis detection kit containing Annexin V, fluorescein isothiocyanate (FITC), and PI was used. Fig. 6B shows that cells treated with AZB-CF_3_@DSPE-PEG followed by light irradiation encountered apoptosis at a rate of 16%, which is higher than cells not treated with light (7%).

These findings indicated that the dye, even after forming NPs, retained its light activity and its ability to destroy cancer cells through photothermal mechanisms.

### Photothermal efficiency of AZB-CF_3_ nanoparticles *in ovo*

#### *In ovo* toxicity and anti-angiogenesis study

**Fig. 7:**
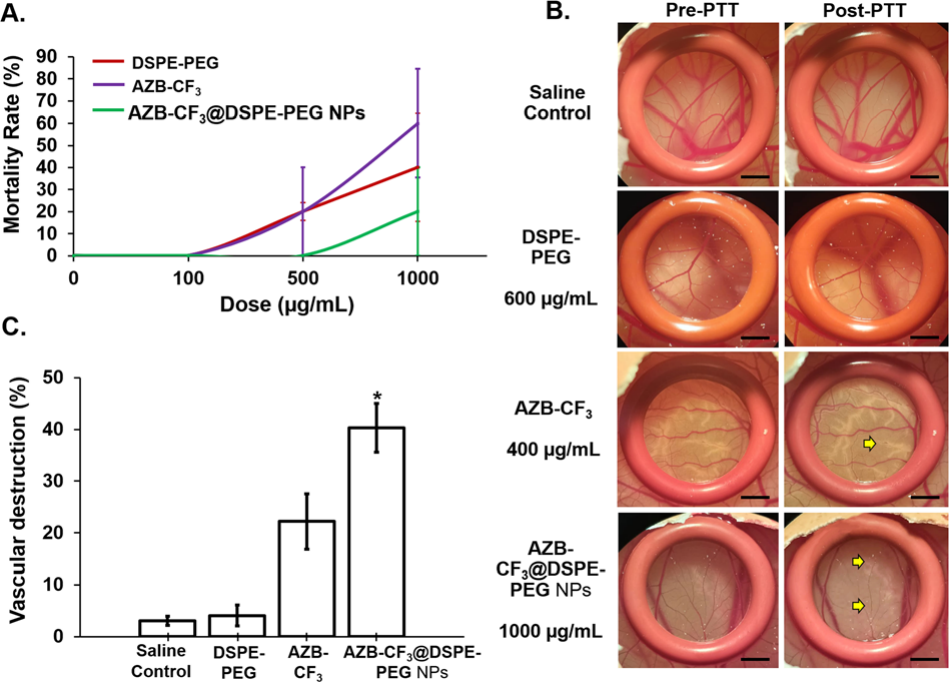
(A) Toxicity profile of AZB-CF_3_@DSPE-PEG NPs, AZB-CF_3_, and DSPE-PEG at concentrations of 100–1000 μg mL^−1^. Data are presented as means ± SEM (*n* = 5). (B) The vasculature of the chorioallantoic membrane in pre- and post-PTT. The yellow arrow indicates the area of blood vessel destruction (scale bar = 20 μm, magnification = 10×). The diagrams shown are representative of each group with similar observations. (C) The percentage of vasculature destruction of all groups at 10 min post-PTT. Data are presented as means ± SEM (*n* = 5), *p < 0.05 based on one-way ANOVA.

The acute toxicity of AZB-CF_3_@DSPE-PEG NPs was determined based on the mortality of the chick embryo at 24 h post-intravenous administration. As shown in Fig. 7A, the LD_50_ of AZB-CF_3_@DSPE-PEG NPs, AZB-CF_3_, and DSPE-PEG on the chick embryo were 1142.12 μg mL^−1^, 882.43 μg mL^−1^, and 1085.64 μg mL^−1^, respectively. From the obtained LD_50_ data, the AZB-CF_3_@DSPE-PEG NPs showed improved biocompatibility as they were found to be less toxic as compared to the parent drug AZB-CF_3_.

Next, the anti-angiogenesis ability of all the samples was compared post-PTT in the chick chorioallantoic membrane (CAM). As shown in Fig. 7B, the CAMs treated with saline and DSPE-PEG at 600 μg mL^−1^ (equivalent to the DSPE-PEG content in AZB-CF_3_@DSPE-PEG NPs at 1000 μg mL^−1^) did not induce any visible morphological changes in the blood vessel post-photo-irradiation. Conversely, both AZB-CF_3_ and AZB-CF_3_@DSPE-PEG NPs-treated CAMs showed vasculature destruction at 10 min post-PTT. AZB-CF_3_-treated CAM at 400 μg mL^−1^ (equivalent to the AZB-CF_3_ content in AZB-CF_3_@DSPE-PEG NPs at 1000 μg mL^−1^) exhibited thinning of blood vessels as compared to pre-PTT, while AZB-CF_3_@DSPE-PEG NPs induced severe vascular damage with a larger area of blood capillary disruption post-PTT.

Quantitatively, saline- and DSPE-PEG-treated CAMs displayed very minor or negligible anti-angiogenic activity of 3.08 ± 0.87% and 4.10 ± 1.98%, respectively. An enhanced and higher anti-angiogenic efficacy was demonstrated by AZB-CF_3_@DSPE-PEG NPs with vascular destruction of 40.47 ± 4.56% after photo-irradiation as compared to the parent drug, AZB-CF_3_ with 22.21 ± 5.34% destruction (*p* = 0.008) (Fig. 7C).

#### Antitumor study

The antitumor efficacy of AZB-CF_3_@DSPE-PEG NPs was evaluated *in ovo* using murine 4T1-implanted tumor xenograft in CAMs. The temperature of the tumor tissue was recorded during the photo-irradiation. A slight increase in the localized tumor temperature (2–3 °C) in AZB-CF_3_-treated CAM was observed at 10–30 s of irradiation. AZB-CF_3_@DSPE-PEG NPs-treated CAM also demonstrated an increase in the temperature (approx. 3–3.5 °C) at 10–30 s of irradiation as compared to pre-irradiation and this was maintained throughout 60 s of irradiation, which is higher than the AZB-CF_3_. The temperatures in the control saline and DSPE-PEG-treated CAM were maintained throughout irradiation as compared to pre-irradiation (Fig. S14†).

**Fig. 8:**
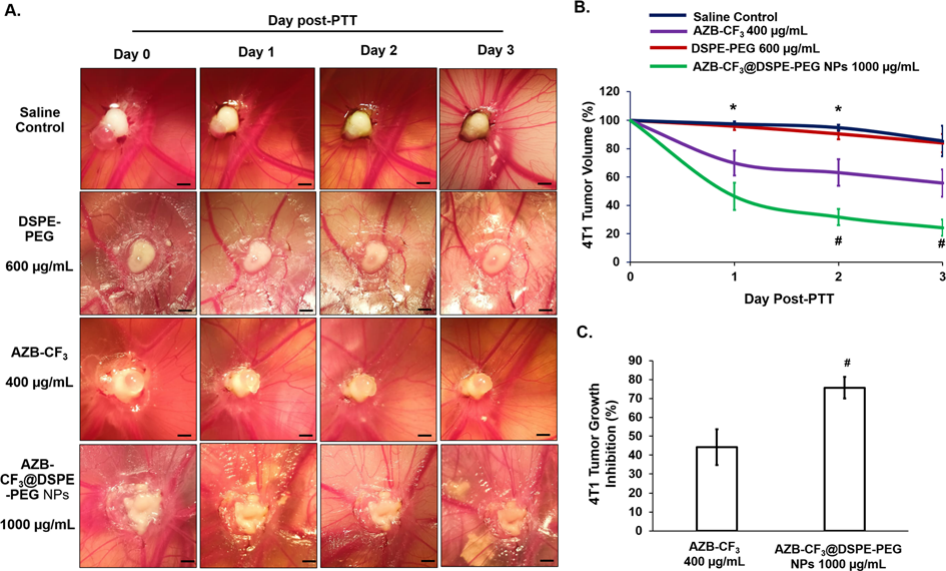
(A) Murine 4T1 tumor in CAM pre- (day 0) and post-PTT (days 1, 2, and 3). Scale bar = 20 μm. The diagrams shown are representative of each group, with the same egg for 3 days of observation. (B) The percentage of 4T1 tumor volume changes across 3 days of observation. Data are presented as means ± SEM (*n* = 5), *p < 0.05, saline-treated control *vs.*AZB-CF_3_ and AZB-CF_3_@DSPE-PEG NPs; #*p* < 0.05, AZB-CF_3_*vs.*AZB-CF_3_@DSPE-PEG NPs based on one-way ANOVA. (C) The percentage of *in ovo* 4T1 tumor growth inhibition at day 3 post-PTT. Data are presented as means ± SEM (*n* = 5), #*p* < 0.05, AZB-CF_3_*vs.*AZB-CF_3_@DSPE-PEG NPs.

The tumor tissue in the CAM post-irradiation was observed daily, for 72 h. As shown in Fig. 8A, the saline- and DSPE-PEG-treated CAMs post-PTT showed comparable tumor volumes with pre-PTT, up to 72 h. Comparatively, AZB-CF_3_ and AZB-CF_3_@DSPE-PEG NPs-treated CAMs showed a significant decline in tumor volume as compared to pre-PTT.

The tumor growth curve was plotted across 3 days post-PTT. A comparable tumor volume was observed in both saline- and DSPE-PEG-treated CAMs as compared to pre-PTT. At an equivalent dose, AZB-CF_3_- and AZB-CF_3_@DSPE-PEG NPs-treated CAMs showed 44.19 ± 9.56% and 75.72 ± 5.75% reduction in the tumor volume, respectively, at 72 h post-PTT (*p* = 0.044) (Fig. 8B and C). This suggests that AZB-CF_3_@DSPE-PEG NPs have better efficacy in inhibiting tumor growth, which might be due to the accumulation of AZB-CF_3_@DSPE-PEG NPs at the tumor site by passive targeting. Similarly, the aza-BODIPY-derived probe (CB1) was synthesized and encapsulated with DSPE-PEG_2000_ by Yang *et al.* and showed an enhanced photothermal effect, inducing significant antitumor activity in the 4T1 grafted mice model as compared to the parent drug CB1.^56^ Other polymeric aza-BODIPY nanoparticles, such as an iodine-substituted aza-BODIPY (B4) coated with DSPE-PEG_5000_,^22^ an aza-BODIPY-based phototherapeutic agent (B-3) encapsulated with DSPE-PEG_5000_ and F108,^57^ and an aza-BODIPY with dimethylamino moieties (A1) constructed with DSPE-PEG_5000_,^28^ exhibited enhanced photothermal antitumor effects *in vivo*. These reports further prove the enhancement of the therapeutic outcome in the presence of nanocarriers.

#### Tumor-tissue staining and anti-metastasis study

**Fig. 9:**
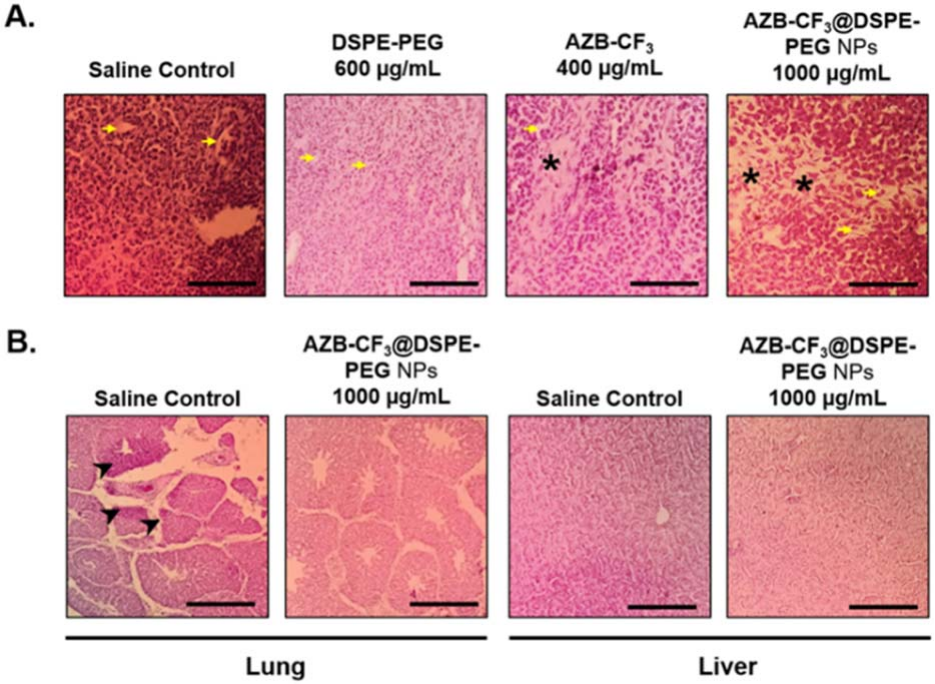
(A) Hematoxylin and eosin-stained 4T1 tumor tissue at day 3 post-PTT. The yellow arrow indicates blood vessels, and the asterisk indicates the tumor necrotic area (scale bar = 80 μm, magnification = 400×). (B) Hematoxylin and eosin-stained lung and liver tissues at day 3 post-PTT. The arrowhead indicates 4T1 murine mammary carcinoma metastasis (scale bar = 20 μm, magnification 100×).

To further evaluate the therapeutic effect and confirm that the decrease in the tumor volume is due to PTT-induced tumor necrosis, hematoxylin and eosin staining of the tumor tissue was conducted. As shown in Fig. 9A, tumor tissues from both the saline-treated and DSPE-PEG-treated CAMs were densely packed and tumor cells were intact with no noticeable cellular damage. However, regional tumoral necrosis indicated by loss of nucleus and cell numbers was observed in both groups treated with AZB-CF_3_ and AZB-CF_3_@DSPE-PEG NPs, where the AZB-CF_3_@DSPE-PEG NPs-treated group showed the larger area of tumor necrotic regions. The necrotic tumor tissue was surrounded by blood vessels (yellow arrow), thus suggesting the efficient delivery of the drug into the tumor microenvironment leading to cell death and necrosis after photoirradiation.

AZB-CF_3_@DSPE-PEG NPs were further evaluated in the 4T1 xenografted CAMs model for preventing spontaneous metastasize to the lung. Histopathological analysis of the lung tissue revealed that the topically xenografted murine 4T1 mammary carcinoma cells had metastasized to the lung of the developing chick embryo in the saline-treated CAMs, as indicated by the accumulation of neoplastic epithelial cells (arrowhead). However, this phenomenon is absent in the AZB-CF_3_@DSPE-PEG NPs-treated CAMs (Fig. 9B). This result confirms that AZB-CF_3_@DSPE-PEG NPs-treated CAMs post-PTT effectively shut down the vessels, reducing tumor volume and hence preventing the spread of the tumor to distant organs.

The liver is also the main detoxifying organ where the metabolism of drugs takes place, hence, liver tissue was evaluated histologically and compared between the saline- and AZB-CF_3_@DSPE-PEG NPs-treated CAMs. Normal liver morphology was observed in the AZB-CF_3_@DSPE-PEG NPs-treated CAMs, suggesting that the metabolism of the NPs did not induce cellular damage to the liver in the chick embryo (Fig. 9B). Altogether, these findings highlight the potential of AZB-CF_3_@DSPE-PEG NPs as a multifunctional photothermal cancer therapeutic by effectively inhibiting angiogenesis, cancer growth, and distant metastasis with remarkable biocompatibility.

## Conclusions

A photothermal-based aza-BODIPY derivative (AZB-CF_3_) has been successfully synthesized and encapsulated into nanoparticles *via* the nanoprecipitation method. The obtained NPs exhibited a round shape with an average hydrodynamic size of 70 nm, as well as a broad Vis-NIR absorbance with a maximum at 870 nm. Although AZB-CF_3_ showed near-infrared absorption in a lipophilic environment, the formulated nanoparticles were non-fluorescent in aqueous media due to an aggregation-caused quenching phenomenon. In addition, the AZB-CF_3_@DSPE-PEG NPs exhibited great photostability and colloidal stability, good biocompatibility *in vitro* and *in ovo*, and high photothermal (PTT) efficacy, which are suitable for PTT-based cancer treatment. In the cell-based evaluation, the NPs (20 μM AZB-CF_3_ content) combined with 5 min of 808 nm laser irradiation led to about 10% viability of 4T1 breast cancer cells. The additional proof in the chicken egg 4T1-tumor model suggested that the AZB-CF_3_@DSPE-PEG NPs are good candidates for photothermal cancer therapeutic agents as they display excellent antitumor efficiency, anti-angiogenesis, and anti-metastasis properties.

## Conflicts of interest

There are no conflicts to declare.

## Supplementary Material

NA-006-D3NA00718A-s001
