# Supplementary material for: Aza-BODIPY-based polymeric nanoparticles for photothermal cancer therapy in a chicken egg tumor model
Source: Nanoscale Adv. 2023 Oct 27;6(2):406–17. doi: 10.1039/d3na00718a (PMC10790972; doi:10.1039/d3na00718a)
Supplement: NA-006-D3NA00718A-s001 [file NA-006-D3NA00718A-s001.pdf]

Electronic Supplementary Material (ESI) for Nanoscale Advances.
This journal is © The Royal Society of Chemistry 2023

Supporting Information

Aza-BODIPY based Polymeric Nanoparticles for Photothermal Cancer Therapy in Chicken Egg Tumor Model

Kantapat Chansaenpak[a]*, Gong Yi Yong[b], Anawin Prajit[c], Peraya Hiranmartsuwan[a], Shaamini Selvapaandian[b], Bongkot Uengwanarat [c], Tunyawat Khrootkaew[c], Piyanut Pinyou[c], Chin Siang Kue[b]*, and Anyanee Kamkaew[c]*

[a] National Nanotechnology Center, National Science and Technology Development Agency, Thailand Science Park, Pathum Thani, Thailand 12120

[b] Faculty of Health and Life Sciences, Management and Science University, Seksyen 13, Shah Alam, Selangor, Malaysia 40100

[c] School of Chemistry, Institute of Science, Suranaree University of Technology, Nakhon Ratchasima, Thailand 30000

*E–mail: kantapat.cha@nanotec.or.th (Kantapat Chansaenpak, Corresponding author)

cskue@msu.edu.my (Chin Siang Kue, Corresponding author)

anyanee@g.sut.ac.th (Anyanee Kamkaew, Corresponding author)

Page

^{1}H and ^{13}C Nuclear Magnetic resonance (NMR) spectra.......2

Mass spectrometry results.......5

Photophysical property measurements.......7

Calculation of the photothermal conversion efficiency.......9

Details on finding NP characteristics.......11

Photothermal effect in tumor tissue.......14

Photothermal conversion efficiencies of aza-BODIPY based nanoparticle reported in the literature.....15

Cyclic voltammogram.......16

1. ^{1}H and ^{13}C Nuclear Magnetic resonance (NMR) spectra

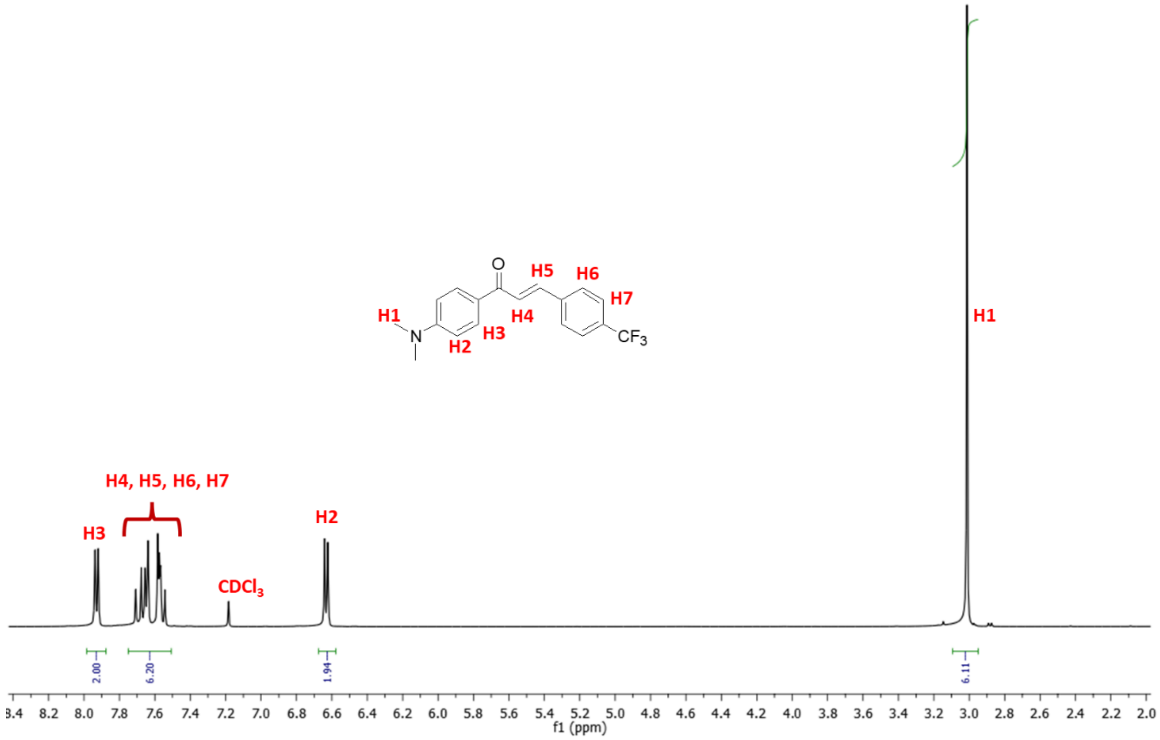

Figure S1. ^{1}H NMR spectrum of **1**.

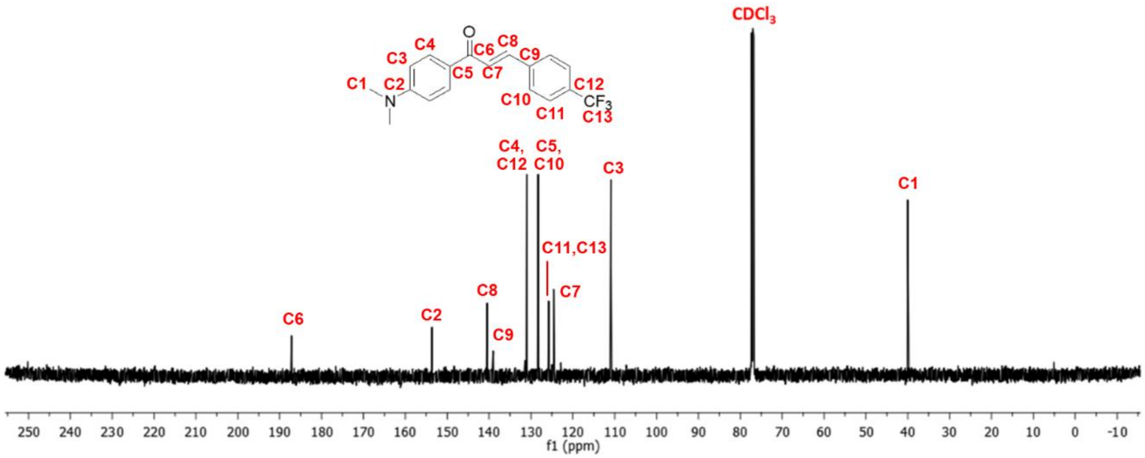

Figure S2. ^{13}C NMR spectrum of **1**.

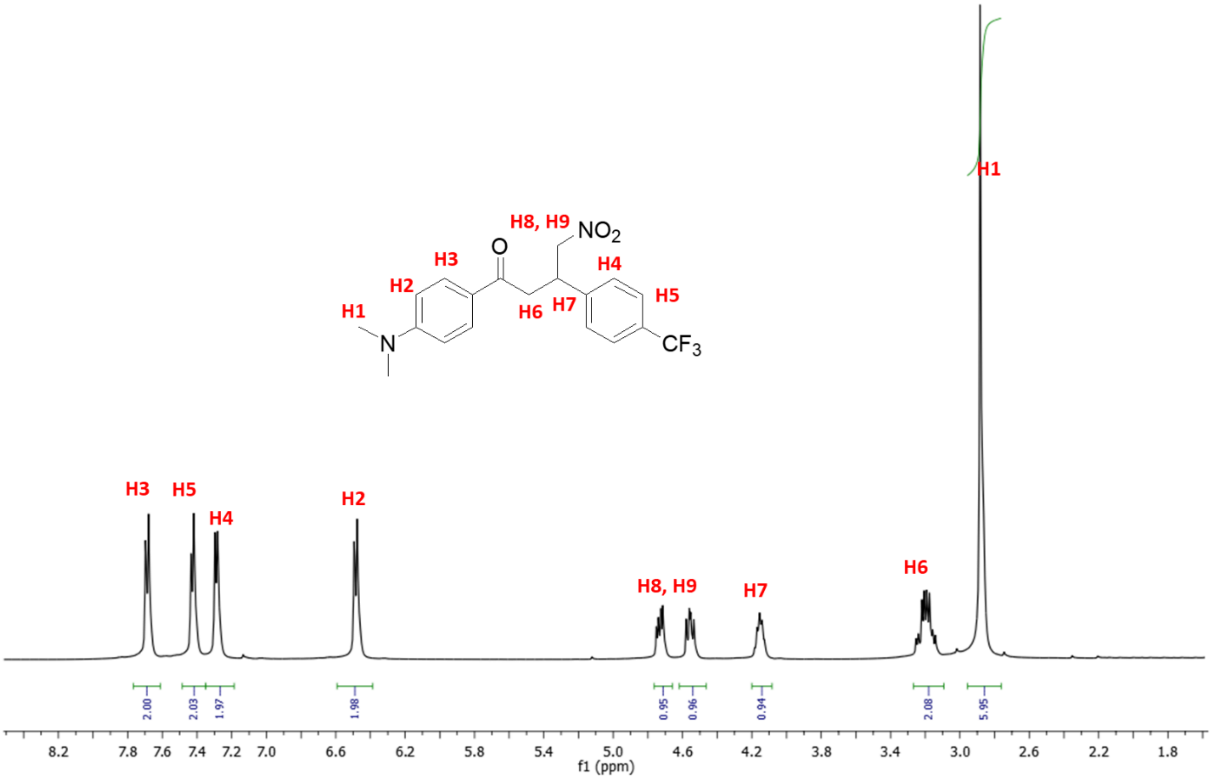

Figure S3. ^{1}H NMR spectrum of **2**.

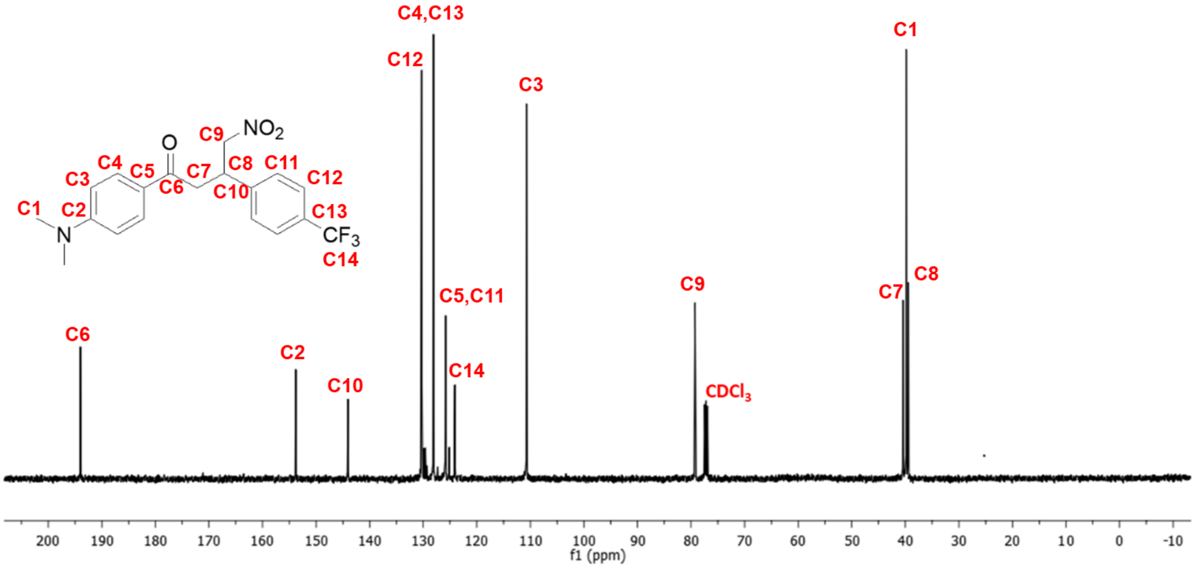

Figure S4. ^{13}C NMR spectrum of **2**.

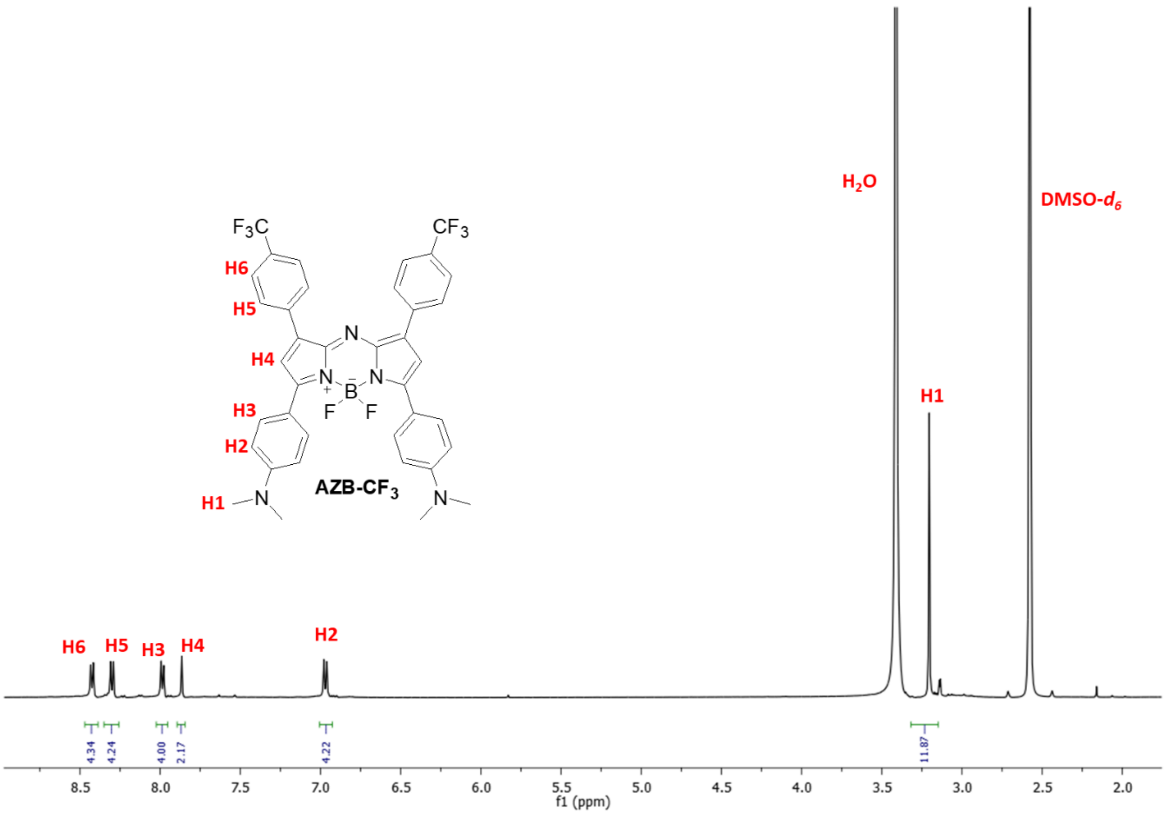

Figure S5. ^{1}H NMR spectrum of **AZB-CF$_3$**.

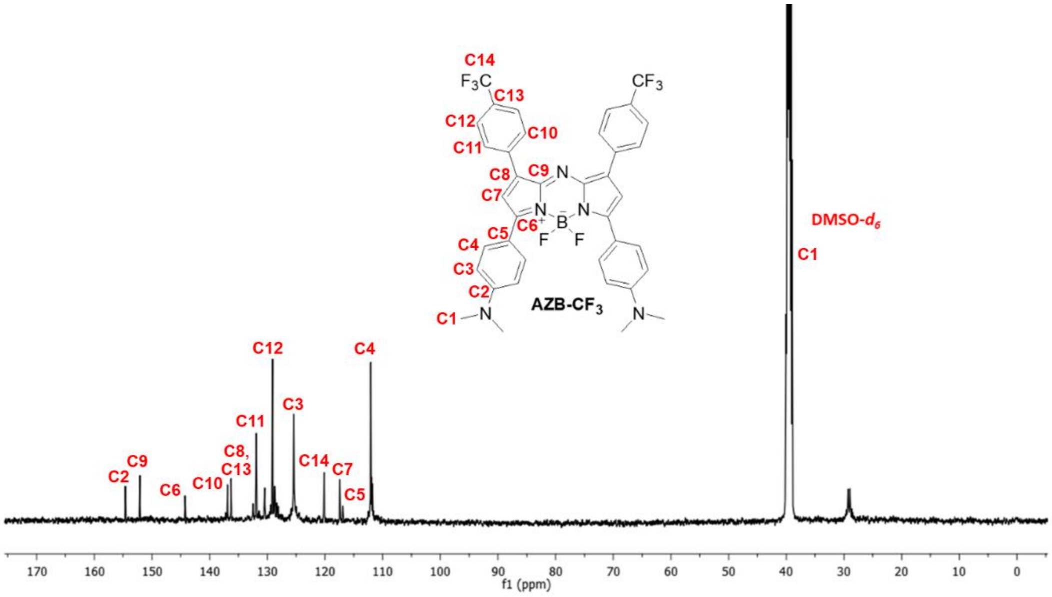

Figure S6. ^{13}C NMR spectrum of **AZB-CF$_3$**.

2. Mass spectrometry results

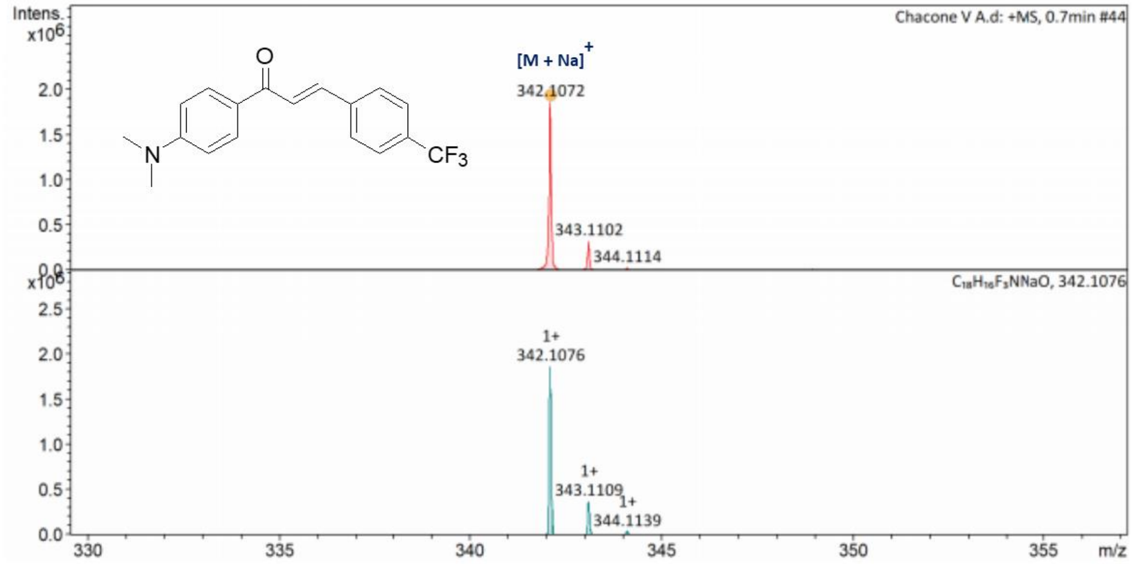

Figure S7. HRMS-ESI of **1**.

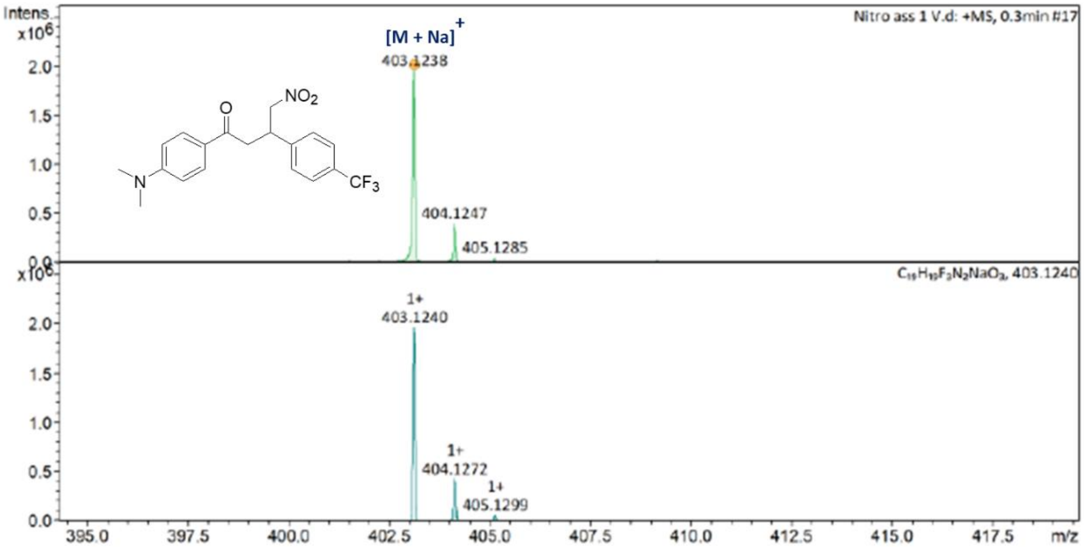

Figure S8. HRMS-ESI of **2**.

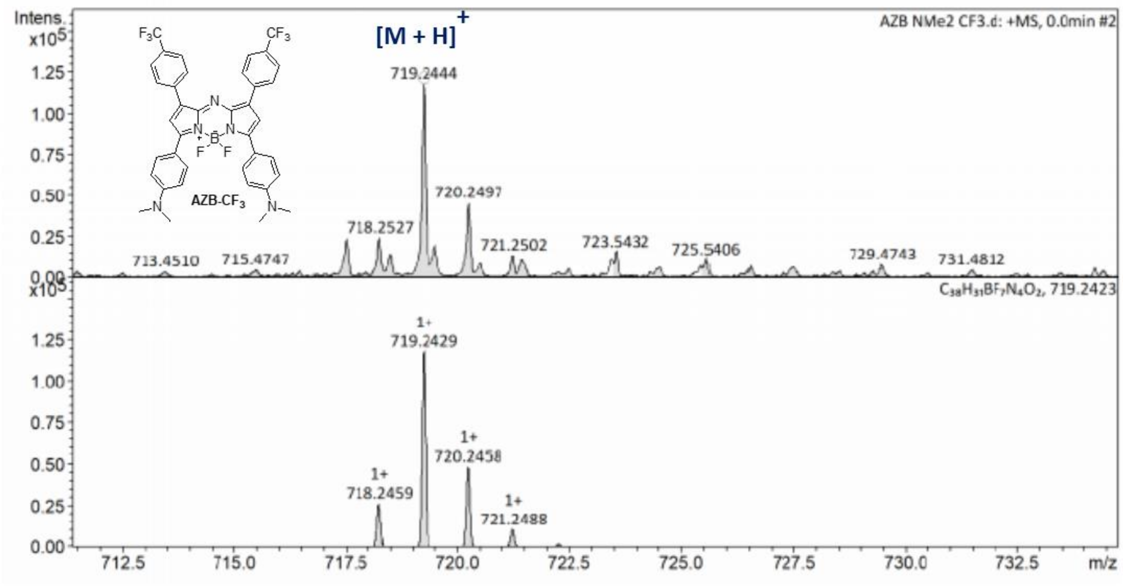

Figure S9. HRMS-ESI of **AZB-CF$_3$**.

3. Photophysical property measurements

General details for UV-Vis and fluorescence measurements and quantum yield calculations

The stock solutions of **AZB-CF_3** derivatives were prepared by dissolving **AZB-CF_3** powders (2.6 mg) with organic solvents in a 3 mL standard volumetric flask (~1.2 x 10^{-3} M). The stock solutions of the nanoparticles were prepared by suspending 2 mg of **AZB-CF_3@DSPE-PEG** nanoparticles in DI water in a 25 mL standard volumetric flask. The UV-Vis and fluorescence measurements were performed by taking an appropriate amount of these stock solutions.

UV-Vis absorption measurement: The stock solution of **AZB-CF_3** derivatives (10 µL) was added to 3.0 mL of organic solvents in a 3.5 mL quartz cuvette (final concentration ~ 4.0 µM). The stock solution of **AZB-CF_3@DSPE-PEG** nanoparticles (3 mL) was added to a 3.5 mL quartz cuvette. The UV-Vis absorption spectra were recorded by a Cary Series UV-Vis-NIR spectrophotometer (Agilent Tech, Santa Clara, CA, USA).

Fluorescence measurement: The stock solution of **AZB-CF_3** derivatives (10 µL) was added to 3.0 mL of organic solvents in a 3.5 mL quartz cuvette (final concentration ~ 4.0 µM). The fluorescence spectra were recorded by a PTI QuantaMaster 500 – Near Infra-Red Photoluminescence System (HORIBA Scientific) using the following parameters: excitation wavelengths = 810 nm, excitation slit widths = 10 nm, and emission slit widths = 10 nm.

The fluorescence quantum yields (Φ_f) which define as the ratio of the number of photons emitted to the number of photons absorbed were calculated using the equation (S1) and relating to Indolecyanine green (ICG) in DMSO (Φ_f = 0.13).

$$\Phi_f = \Phi_{std} \times \left(\frac{A_{sample}}{A_{std}}\right) \times \left(\frac{I_{std}}{I_{sample}}\right) \times \left(\frac{\eta_{sample}}{\eta_{std}}\right)^2 \quad \text{................} \ (S1)$$

where Φ denotes fluorescence quantum yield, *A* is a peak area of emission, *I* is an absorbance at the excitation wavelength, and *η* stands for solvent reflective index.

Singlet Oxygen Quantum Yields (Φ_Δ)

Singlet oxygen quantum yields of **AZB-CF_3@DSPE-PEG** and **AZB-CF_3** were determined using a laser (808 nm, power density of 1 W cm^{-2}) in PBS buffer pH 7.4 at room temperature compared to a standard (ICG). A solution containing 20 µM of 1,3-

diphenylisobenzofuran (DPBF) as a singlet oxygen scavenger and 1 µM of **AZB-CF₃@DSPE-PEG** in a quartz cell of 1 cm path length. The DPBF solution in PBS buffer pH 7.4 (negative control), the solution containing 1 µM of **AZB-CF₃**, and the solution containing 1 µM ICG (comparative control) were also examined. After being exposed to the laser, the absorbance reduction of DPBF at 408 nm was measured for 1-10 min by a Shimadzu UV-Vis spectrophotometer (UV-1900i). The changing of absorbance is plotted against irradiation time. The singlet oxygen quantum yield was calculated according to equation (S2),

$$\Phi_{\Delta} = \Phi_{std}(\frac{grad_{sample}}{grad_{std}})(\frac{F_{std}}{F_{sample}}) \qquad \text{(S2)}$$

where Φ_{std} denotes the singlet oxygen quantum yield of ICG (0.077 in DMSO), grad is the rate of reaction, and F is the absorption correction factor ($F = 1{-}10^{-\text{absorbance}}$).

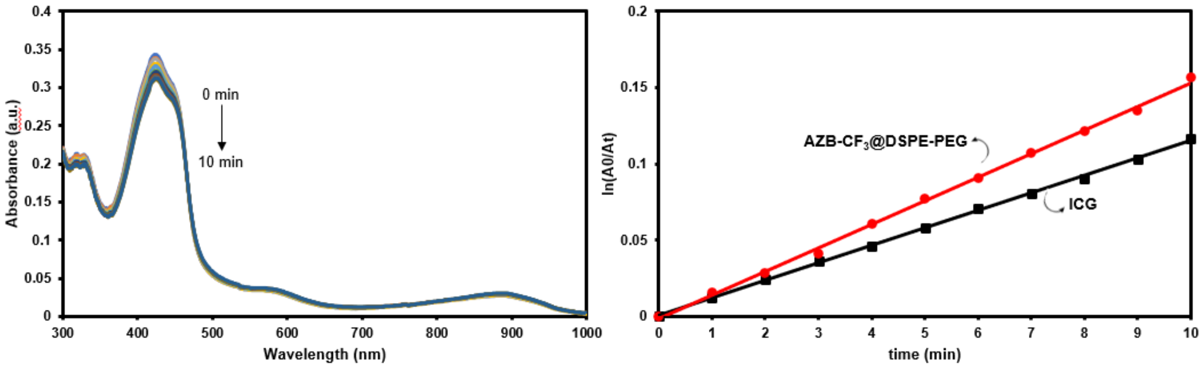

Figure S10. (A) Changes in the absorption spectra of DPBF upon irradiation in the presence of **AZB-CF₃@DSPE-PEG** from 0 to 10 min (recorded at 60 s intervals). (B) A plot of changes in absorbance of DPBF at 418 nm *vs* irradiation time (λ_{irr} = 808 nm) in the presence of **AZB-CF₃@DSPE-PEG** against **ICG** as the standard.

4. Calculation of the photothermal conversion efficiency

The photothermal conversion efficiency (η) of the **AZB-CF$_3$@DSPE-PEG** was calculated according to:

$$\eta = \frac{hs(T_{Max}-T_{Surr})- Q_{Dis}}{I(1-10^{-A_{808}})} \text{..........................(S3)}$$

Where *h* is the heat transfer coefficient, *s* is the surface area of the container and the value of *hs* is obtained from Eq. S6 and **Figure S13**. The maximum steady temperature (T_{max}) of the solution of the **AZB-CF$_3$@DSPE-PEG** was 79.1 °C and the environmental temperature (T_{Surr}) was 30.7 °C. So, the temperature change (T_{Max}-T_{Surr}) of the solution of the **AZB-CF$_3$@DSPE-PEG** was 48.4 °C. The laser power *(I)* is 1.0 W. The absorbance of the **AZB-CF$_3$@DSPE-PEG** at 808 nm A_{808} is 0.944. Q_{Dis} expresses heat dissipated from the light absorbed by the solvent and container.

To gain *hs*, a dimensionless parameter (Θ) is introduced as followed:

$$\Theta = \frac{T- T_{Surr}}{T_{Max}- T_{Surr}} \text{..............................(S4)}$$

A sample system time constant (τ_s) can be calculated from Eq.3 using data from **Figure S11**.

$$t = -\tau_s \ln(\Theta) \text{..............................(S5)}$$

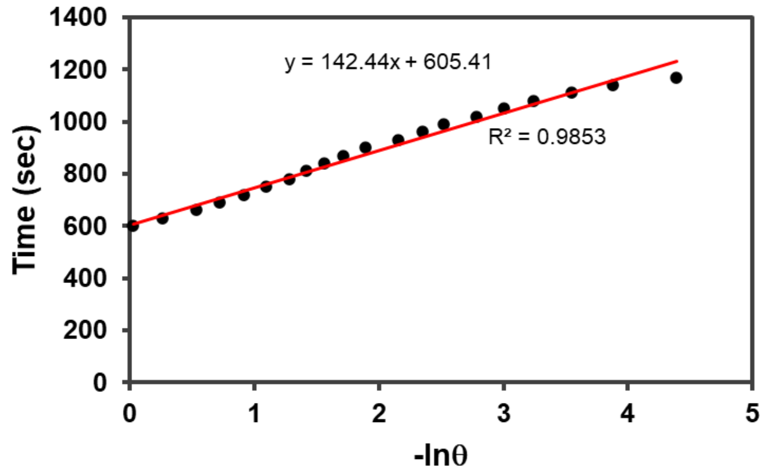

Figure S11. Linear time data versus -ln(θ) obtained from the cooling period in **Figure 6B**.

According to Figure S11, τ_s was determined as 142.44 s.

$$hs = \frac{mC}{\tau_s} \text{....................................(S6)}$$

In addition, m is 0.3 g and C is 4.2 J/g·°C. Thus, according to Eq. 4, *hs* is deduced to be 0.00885 mW/°C.

Q_{Dis} expresses heat dissipated from the light absorbed by the quartz sample cell itself, and it was measured independently to be 0.0304 mW using a quartz cuvette cell containing pure water.

Thus, according to values of each parameter in Eq. S5, the 808 nm laser heat conversion efficiency (η) of the **AZB-CF_3@DSPE-PEG** was calculated as 44.9%.

5. Details on finding NP characteristics

Determinations of encapsulation efficiency (%EE)

Encapsulation efficiency (%EE) defined as a percentage of the **AZB-CF_3** dye that has been successfully encapsulated into the NPs to the total amount of dye feed was calculated based on equation (S7). To determine the %EE of **AZB-CF_3**, the calibration curves were first constructed by plotting the absorbance at 830 nm of **AZB-CF_3** dye in CH_2Cl_2 solutions at different concentrations of **AZB-CF_3**. The freeze-dried **AZB-CF_3@DSPE-PEG** NPs were then dissolved in CH_2Cl_2, and the absorbances at 830 nm were recorded by UV-Vis-NIR spectrophotometer. The amount of **AZB-CF_3** encapsulated in the NPs was determined from the standard calibration curve to calculate the encapsulation efficiency based on equation (S7).

$$\left(\frac{Amount\ of\ dye\ encapsulated\ in\ NPs}{Amount\ of\ dye\ feed}\right) \times 100 \quad \text{…………………}(S7)$$

Determinations of dye loading percentage

Dye-loading percentages were determined from the ratio of the mass of the encapsulated dye to the total mass of NPs as shown in equation (S8). To obtain the dye-loading percentages in NPs, the freeze-dried **AZB-CF_3@DSPE-PEG** NPs were weighted to get the total mass of NPs prior to dissolving in CH_2Cl_2. Then, the mass of **AZB-CF_3** encapsulated in NPs was determined from the standard calibration curve.

$$\left(\frac{mass\ of\ dye\ encapsulated\ in\ NPs}{Total\ mass\ of\ dried\ NPs}\right) \times 100 \quad \text{…………………..}\ (S8)$$

Colloidal stability test

The colloidal stability test of **AZB-CF_3@DSPE-PEG** NPs was carried out in 0.1 M phosphate buffer solution (PBS), pH 7.4. The hydrodynamic sizes (DLS sizes) and zeta potentials of the NPs were recorded by Zetasizer Nano series (Malvern Panalytical) at day 1, 3, 5, 8, and 13 after incubation in PBS (n = 5).

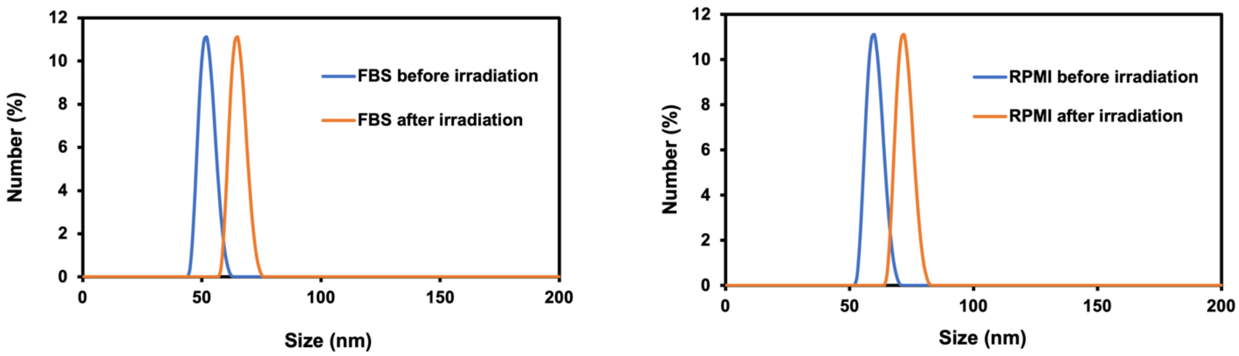

NPs	Irradiation	Zeta Potential (mV)
AZB-CF_3 NPs in RPMI	before	-3.82
AZB-CF_3 NPs in RPMI	after	-3.51
AZB-CF_3 NPs in FBS	before	-3.07
AZB-CF_3 NPs in FBS	after	-2.55

Figure S12. Hydrodynamic size and zeta potential of **AZB-CF_3@DSPE-PEG** NPs in Roswell Park Memorial Institute (RPMI) 1640 culture medium and fetal bovine serum (FBS), before and after light irradiation.

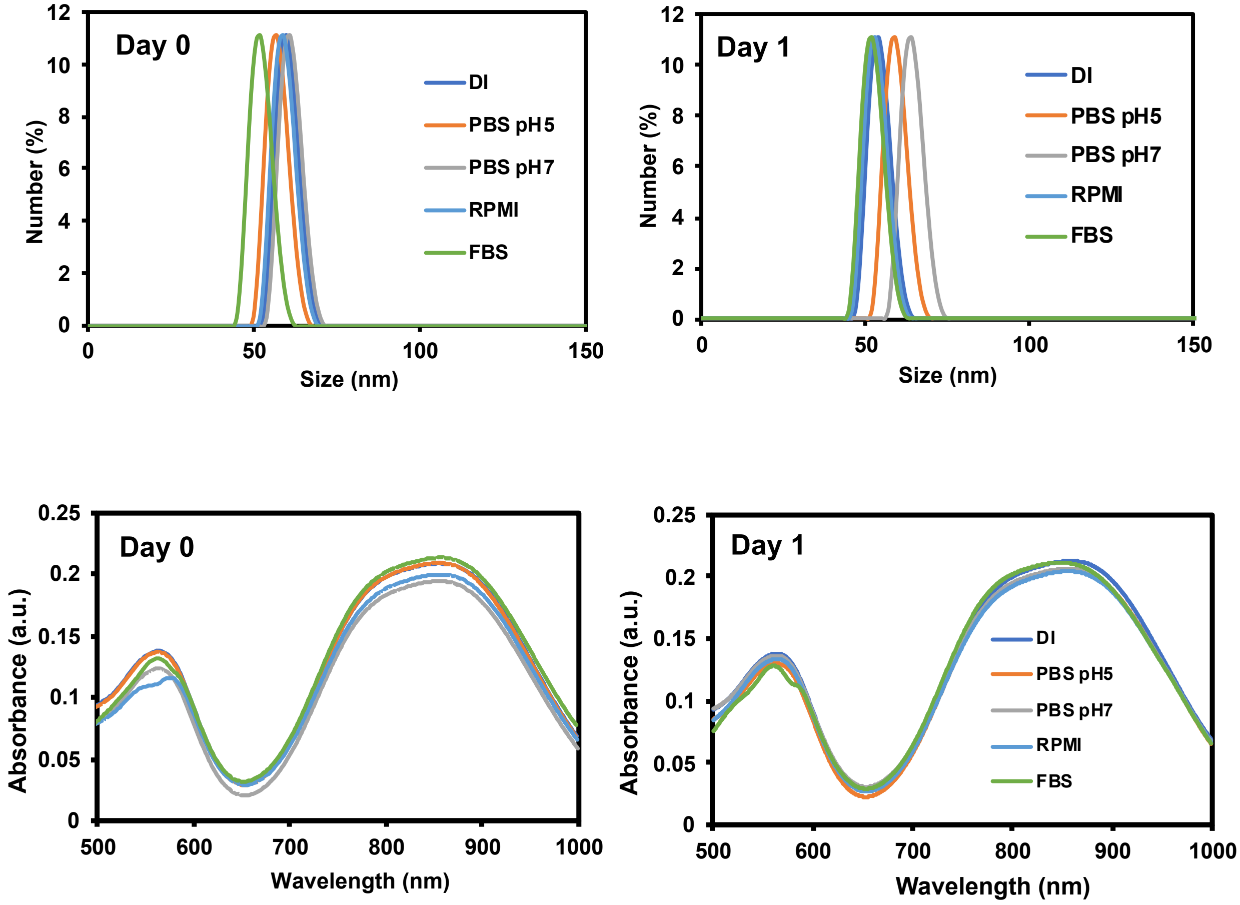

Figure S13. Hydrodynamic sizes from DLS and Vis-NIR absorption spectra of **AZB-CF_3@DSPE-PEG** NPs in various solutions.

6. Photothermal effect in tumor tissue

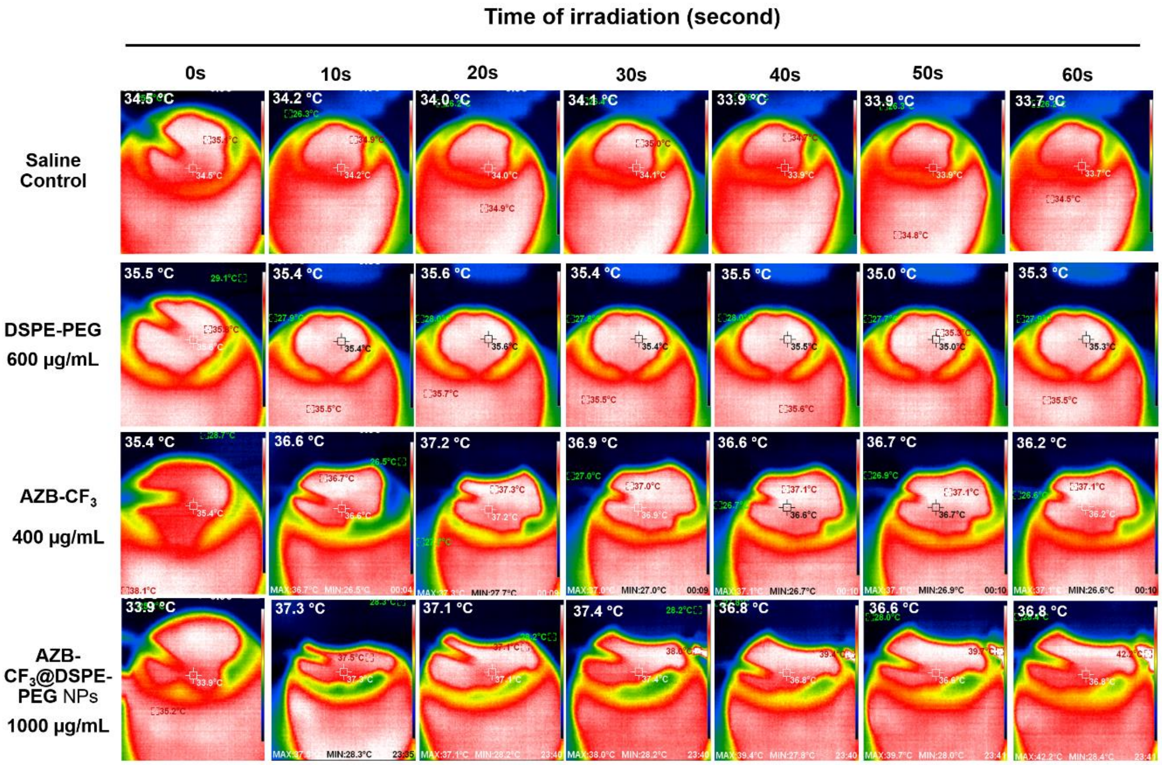

Figure S14. The temperatures of the tumor tissue (white font) at different time points throughout 1 minute of irradiation.

Table S1. Photothermal conversion efficiencies of aza-BODIPY based nanoparticle reported in the literature.

Structure	Polymeric nanocarrier	Laser (nm)	Photothermal conversion efficiency	Reference
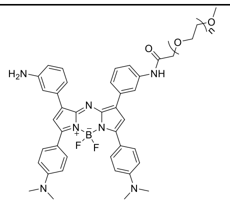	mPEG	808	41.4	ACS Appl. Bio Mater. 2022, 5, 9, 4567–4577
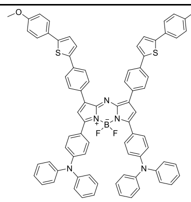	$POEGMA_{23}$–$PAsp_{20}$	915	35.6	J. Mater. Chem. B, 2022,10, 1650-1662
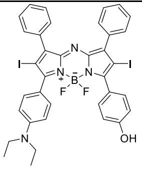	PDPA-PEG	730	40.17	Nano Res. 15, 2022, 716–727
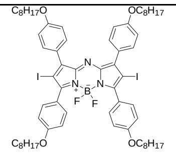	DSPE-mPEG	730	34.8	Adv. Healthcare Mater. 2018, 7, 1800606
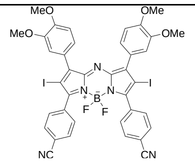	mPEG	660	38.3	Adv. Healthcare Mater. 2018, 7, 1701272
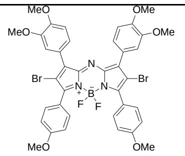	mPEG	730	40.0	J. Mater. Chem. B, 2018,6, 4522-4530
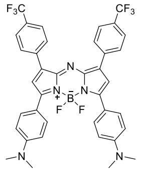	DSPE-mPEG	808	44.9	This work

7. Cyclic voltammogram

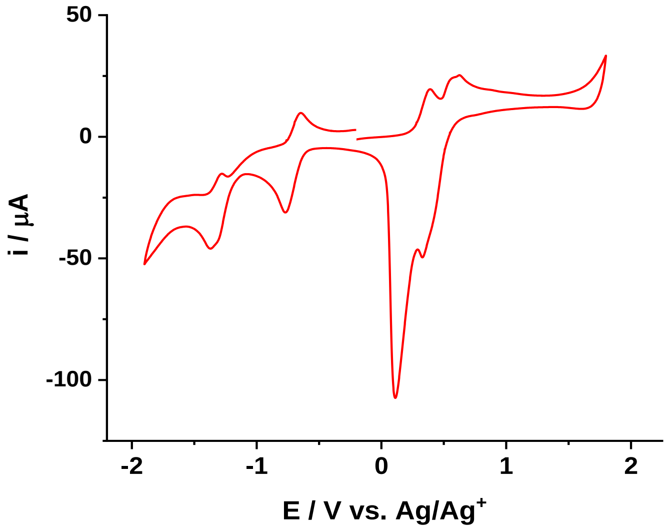

Figure S15. Cyclic voltammogram of 1.4 mM **AZB-CF$_3$** in CH_2Cl_2 containing 0.1 M TBAPF$_6$ at the scan rate of 0.100 V/s
